# Supplementary material for: Extracting nanoscale membrane morphology from single-molecule localizations
Source: Biophys J. 2023 Jun 23;122(15):3022–30. doi: 10.1016/j.bpj.2023.06.010 (PMC10432223; doi:10.1016/j.bpj.2023.06.010)
Supplement: Document S2. Article plus supporting material [file mmc3.pdf]

Biophysical Journal
Computational Tool

Biophysical Society

Extracting nanoscale membrane morphology from single-molecule localizations

Zach Marin,[1,2,3] Lukas A. Fuentes,[2] Joerg Bewersdorf,[2,3,4] and David Baddeley[1,2,*]
[1]Auckland Bioengineering Institute, University of Auckland, Auckland, New Zealand; [2]Department of Cell Biology, Yale University School of Medicine, New Haven, Connecticut; [3]Department of Biomedical Engineering, Yale University, New Haven, Connecticut; and [4]Department of Physics, Yale University, New Haven, Connecticut

ABSTRACT Membrane surface reconstruction at the nanometer scale is required for understanding mechanisms of subcellular shape change. This historically has been the domain of electron microscopy, but extraction of surfaces from specific labels is a difficult task in this imaging modality. Existing methods for extracting surfaces from fluorescence microscopy have poor resolution or require high-quality super-resolution data that are manually cleaned and curated. Here, we present NanoWrap, a new method for extracting surfaces from generalized single-molecule localization microscopy data. This makes it possible to study the shape of specifically labeled membranous structures inside cells. We validate NanoWrap using simulations and demonstrate its reconstruction capabilities on single-molecule localization microscopy data of the endoplasmic reticulum and mitochondria. NanoWrap is implemented in the open-source Python Microscopy Environment.

SIGNIFICANCE We introduce a novel tool for reconstruction of subcellular membrane surfaces from single-molecule localization microscopy data and use it to visualize and quantify local shape and membrane-membrane interactions. We benchmark its performance on simulated data and demonstrate its fidelity to experimental data.

INTRODUCTION

Changes in cellular membrane shape are linked to viral replication, Alzheimer's disease, heart disease, and an abundance of other maladies (1–7). Understanding the morphological mechanisms behind these diseases requires imaging not only the locations of protein clusters causing structural changes in a membrane, but finding the underlying shape of the membrane at relevant size scales. Some membranous organelles, such as the endoplasmic reticulum (ER) and the Golgi, have diameters as small as ~ 50 nm, requiring an image resolution of 25 nm or better to properly resolve structure (8,9). Electron microscopy (EM) techniques have a resolution of 2 nm or better and are well suited to imaging membranes. However, segmentation of membrane structures from EM images can be arduous (10,11) and it can be difficult to label and identify specific proteins in EM samples. Immunolabeling with gold nanoparticles, the most common method for specifically labeling proteins in EM samples, requires fixation methods that often destroy cellular structures and suffers from relatively poor labeling efficiencies. It also appears in the same channel as all other cellular features, requiring nontrivial image postprocessing to extract the locations of individual gold nanoparticles (12, 13).

Fluorescence microscopy techniques provide spectral separation of multiple fluorescent labels, allowing for easy identification of both membrane-associated and membrane-interacting proteins. However, conventional fluorescence microscopy techniques achieve a resolution no better than 250 nm and are therefore unable to visualize membrane curvature at true size scales. Single-molecule localization microscopy (SMLM) techniques, such as PALM, STORM, and PAINT, image the positions of proteins with $\sim 10-20$ nm resolution (14). This is sufficient for imaging membrane structural changes of interest (15). In contrast to EM imaging techniques, which show a continuous membrane, SMLM yields a sparse and noisy point cloud of fluorophore locations, each with an uncertainty

Submitted March 8, 2023, and accepted for publication June 15, 2023.
*Correspondence: d.baddeley@auckland.ac.nz
Zach Marin and Lukas A. Fuentes contributed equally to this work.
Editor: Sarah Veatch.
https://doi.org/10.1016/j.bpj.2023.06.010
© 2023 Biophysical Society.
This is an open access article under the CC BY license (http://creativecommons.org/licenses/by/4.0/).

that depends on the brightness of the underlying blinking event. To visualize and quantify a membrane, it is necessary to interpolate a continuous surface through these positions.

In the fields of remote sensing (16) and three-dimensional (3D) scanning (17), screened Poisson reconstruction (SPR) (18) is often used to extract surfaces from point clouds. SPR reconstruction is designed to follow point locations exactly, giving it high fidelity to collected positions. SPR has been applied to SMLM data (19), but this required extremely high-quality data that were manually curated and preprocessed. Due to labeling inefficiencies and sampling, SMLM data often show holes in large regions of a structure. To paper over these holes, SPR relies on the normals of surrounding point data to estimate the gradient of the original surface. Accurate normals are easily recovered in 3D scanning, but the high background in SMLM confounds standard estimation methods that rely on neighboring points to calculate normals. Fluorescent labels often bind to not only molecules of interest, but to other, nonspecific targets in the sample. This and sample autofluorescence can generate spurious background localizations (20). The stochastic nature of SMLM imaging means each fluorescent molecule may blink multiple times thus appearing as multiple points, each in a slightly different spot. Adhering strictly to all of these points will not necessarily generate an accurate surface approximation of the underlying structure the point cloud represents.

To generate an accurate surface from general SMLM data, it is necessary to account for the localization precision of each point. By weighting each point's influence on surface structure by its precision, a surface is allowed to move away from points with high uncertainty while still adhering to the data. This makes it possible to ignore contributions of poorly localized spots arising from autofluorescence and nonspecific binding. This is the key difference between our approach and other shrink-wrapping routines, which adhere strictly to or generate surfaces at a constant offset from input point clouds (21,22) (see Fig. S1). Shape priors have been used to smooth meshes sensibly over empty areas in point clouds, but these do not discriminate between background and structural points, and standard loss functions that are robust to outliers, such as the one used in (23), do not account for the heavy background noise conditions seen in SMLM data. Zhao et al. created a surface-fitting algorithm that takes pixel noise into account when generating surfaces from voxel-based images, and extended some of the paper's maths to create an expression that uses localization uncertainty to fit SMLM data sets (24). However, the authors did not create an implementation for SMLM surface fitting. To our knowledge, no research group has demonstrated a general method for fitting surfaces to SMLM data that leverages information about localization uncertainties.

Here we present a novel algorithm, which we call NanoWrap, that creates constrained organelle surface representations from SMLM point clouds. NanoWrap incorporates localization uncertainty into its fitting routine and works for any 3D SMLM data set. Smoothing techniques are often applied to extracted surfaces to achieve reasonable shapes (25,26), and such an approach is used in this algorithm. NanoWrap is implemented in PYthon Microscopy Environment Visualize (https://python-microscopy.org) for ease-of-use, integration with additional SMLM acquisition and analysis techniques, and the ability to pass localizations acquired via a user's microscope and software of choice to the algorithm (27).

MATERIALS AND METHODS

Initial/starting mesh

The major steps of NanoWrap are sketched in Fig. 1. The algorithm takes an initial, coarse surface that loosely approximates a point cloud and iteratively refines the structure under point fidelity and curvature constraints.

First, a set of single-molecule localizations are placed in a sparse octree data structure (28). The octree is truncated at a given minimum number of localizations per octree cell (equivalent to a minimum signal/noise ratio—see (29)). This has the effect of dividing the volume into cubic cells with sizes that adapt to local point density. Cells will be large in areas with few localizations, and small in areas that are localization dense. Cells containing fewer than the minimum number of points are not stored. The result is a volumetric data structure that contains the same information as a regularly sampled grid, but requires significantly less memory. The density of localizations in each cell is calculated. The dual marching cubes algorithm is run on these cells with a given threshold density (30). The result is a manifold triangular mesh that separates high- from low-density areas (27). Because SMLM labeling is sparse, a density-based isosurface can only recover a crude representation of the underlying structure. To avoid issues with oversegmentation, the threshold for this starting estimate should be chosen such that the resulting surface lies outside the true membrane surface (i.e., using a lower threshold value than would be used if trying to estimate the surface directly with an isosurface). The following steps will then pull the surface in onto the true membrane location.

Mesh optimization

The initial mesh is refined to achieve high fidelity to the localization input data under a curvature constraint. This is expressed mathematically as a minimization problem:

$$\underset{\mathbf{v}}{\operatorname{argmin}} \left|\left| \frac{1}{\boldsymbol{\sigma}\left(\frac{\mathbf{p}-\mathbb{A}(\mathbf{v})}{2\boldsymbol{\sigma}}+1\right)}(\mathbf{p}-\mathbb{A}(\mathbf{v})) \right|\right|^2 + \lambda^2 \|\mathbb{B}(\mathbf{v})\|^2 \tag{1}$$

where $\mathbf{p}$ is a vector containing localization positions, $\mathbf{v}$ is a vector containing mesh vertex positions, the $\mathbf{p}-\mathbb{A}(\mathbf{v})$ term represents the distances between each localization and the mesh, $\mathbb{B}(\mathbf{v})$ encodes a curvature penalty, λ is a constant controlling the relative weighting of point fidelity and curvature terms and $\boldsymbol{\sigma}$ is a vector containing localization uncertainties. In microscopic imaging, the localization error ellipse $\boldsymbol{\sigma}$ can generally be assumed to be aligned with the Cartesian axes (using the common convention that x and y are the camera axes, and z is along the optical axis of the microscope) and it is possible to consider errors in x, y, and z independently. If our method were applied to methodologies (e.g., LIDAR) where this assumption cannot be made, the method would need to be adapted to use a covariance matrix.

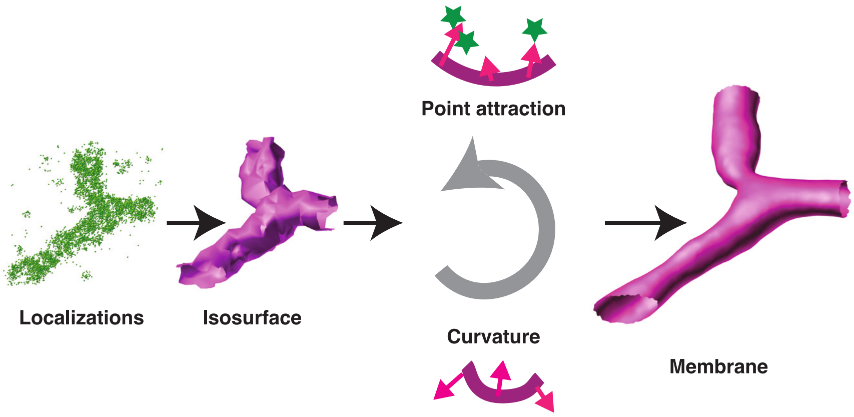

FIGURE 1 A flow diagram representing the major steps of NanoWrap. Localization data are first approximated by a coarse, density-based isosurface. This surface is moved toward the localizations subject to a curvature force constraint. The pipeline runs iteratively until stopping criteria are met. The result is a membrane approximation of the underlying continuous structure sampled by the input localizations. To see this figure in color, go online.

The above equation is iteratively solved using a conjugate gradient method (31). If, after refinement, the mesh does not change shape significantly compared with its shape in the previous iteration, or if the given maximum number of iterations is reached, the algorithm terminates and the resulting surface is presented as an approximation of the underlying structure. Otherwise, the surface is passed to the mesh refinement step and all subsequent steps repeat.

Point fidelity term

Notation.

$\mathbf{p}$ the $N \times 3$ array of fluorophore localizations
$\mathbf{p}_i = \vec{p}_i$ the ith localization (1×3)
$\boldsymbol{\sigma}$ $N \times 3$ array of the uncertainty of the localizations
$\boldsymbol{\sigma}_i$ the uncertainty of the ith localization (1×3)
$\mathbf{v}$ the $M \times 3$ array of mesh vertices
$\mathbf{v}_k = \vec{v}_k$ the kth mesh vertex (1×3)
$\vec{v}_j$ the jth vertex of a given mesh face
$\vec{v}_l$ the lth vertex neighbor of a given vertex

The point attraction term seeks to minimize the distance between each localization and the surface. For each localization, we approximate its distance to the surface as the distance to a proxy point formed by a linear combination of the three vertex positions that define its closest surface face. The weightings used in the linear combination are computed as follows, based on the inverse distance from a localization $\vec{p}_i$ to each vertex $\vec{v}_j$ in its nearest face.

$$w_{ij} = \frac{1}{\left\|\vec{p}_i - \vec{v}_j\left(\vec{p}_i\right)\right\|} \Bigg/ \sum_{j=0}^{2} \frac{1}{\left\|\vec{p}_i - \vec{v}_j\left(\vec{p}_i\right)\right\|} \tag{2}$$

The position of the proxy point is then calculated as

$$\mathbb{A}(\mathbf{v})_i = \sum_{j=0}^{2} \vec{v}_j\left(\vec{p}_i\right) w_{ij}$$

giving the distance metric:

$$[\mathbf{p} - \mathbb{A}(\mathbf{v})]_i = \vec{p}_i - \sum_{j=0}^{2} \vec{v}_j\left(\vec{p}_i\right) w_{ij}.$$

This metric is asymptotically equal to the true distance to the surface at small distances, but trends to the distance between the localization and the center of its nearest face (all vertices weighted equally) at large distances. This behavior was deliberately chosen to ensure sensible updating of vertex positions—for a localization close to the surface we want it to mostly pull on the closest vertex, whereas a point that is far away (compared with the face edge length) should pull equally on all vertices of the face.

This distance metric is weighted by

$$\frac{1}{\boldsymbol{\sigma}_i\left(\frac{[\mathbf{p} - \mathbb{A}(\mathbf{v})]_i}{2\boldsymbol{\sigma}_i} + 1\right)}$$

as shown in Eq. 1. The $\frac{1}{\boldsymbol{\sigma}_i}$ term ensures that distances near a localization produce minimal cost. The remaining fraction deweights localizations that are particularly far away, while still letting them exert influence on the surface. This ensures that the surface can shift to accurately fit all points in a point cloud if necessary, but places priority on moving toward the centroid of its nearby points first. Since localizations are Gaussian distributed, they should exercise most of their influence within $2\boldsymbol{\sigma}_i$ of their position.

Curvature term

When minimizing curvature at a vertex, there are multiple possible formulas to choose from. One of the simplest is the Laplacian discretization of the Canham-Helfrich bending energy (32),

$$E_{\text{bend}}\left(\vec{v}_k\right) = \frac{\kappa}{2}\frac{1}{\Omega_k}\left[\sum_l \vec{v}_k - \vec{v}_l\right]^2$$

where $\Omega_k = \frac{\sqrt{3}}{4}\sum_l \|\vec{v}_k - \vec{v}_l\|^2$, $\vec{v}_l$ is the lth neighbor of vertex $\vec{v}_k$, and κ is the stiffness coefficient for the lipid composition of the membrane. The local bending energy at vertex $\vec{v}_k$ can then found by minimizing

$$\|\mathbb{B}_1(\mathbf{v}_k)\|^2 = \frac{1}{N^2}\left[\sum_l \left(\vec{v}_k - \vec{v}_l\right)\right]^2 = \left[\vec{v}_k - \vec{c}_k\right]^2$$

or the squared distance between a vertex and the centroid of its neighbors ($\vec{c}_k$). This formulation, however, leads to a shrinking of the membrane, with a surface constrained entirely by curvature eventually collapsing to an infinitely small sphere (a valid, but trivial solution to the bending energy minimization problem). As a result, balancing this curvature term with a point-attraction term will always lead to a surface approximation that lies inside the true surface. In practice, the shrinking effect is sufficiently strong that a curvature weighting (λ) low enough for the point attraction to prevent excess shrinkage will lead to an excessively rough surface.

An alternative, area-preserving approach to curvature minimization ($\mathbb{B}_2$) is to penalize the distance between a vertex and a location on the surface of a sphere fit to the vertex's neighboring vertices. $\mathbb{B}_2$ can be approximated as

$$\mathbb{B}_2 = \vec{v}_k - \frac{1}{N}\sum_l \left[\vec{c}_k + \vec{n}_k \frac{\left(\vec{v}_l - \vec{c}_k\right)\cdot\vec{n}_k}{\sqrt{2\vec{n}_k\cdot\vec{n}_l + 1}}\right]$$

$$= \vec{v}_k - \vec{c}_k - \vec{n}_k\frac{1}{N}\sum_l \frac{\left(\vec{v}_l - \vec{c}_k\right)\cdot\vec{n}_k}{\sqrt{2\vec{n}_k\cdot\vec{n}_l + 1}}$$

where $\vec{n}_k$ and $\vec{n}_l$ are the normals of vertex k and its lth neighbor. This parameterization works when the surface is well constrained by localization data, leading to a smooth surface with good affinity to the localizations, but can result in large, static "blebs" in areas where the starting estimate was poor and localizations are especially sparse (see Fig. S2). Our empirical solution is to use $\mathbb{B}_2$ where the influence of localization data is high, smoothly transitioning to the area minimizing approach, $\mathbb{B}_1$, as influence decreases. This gives $\mathbb{B}$ the following form:

$$\mathbb{B} = (1-\alpha)\mathbb{B}_1 + \alpha\mathbb{B}_2$$

$$= \vec{v}_k - \vec{c}_k - \alpha\vec{n}_k\frac{1}{N}\sum_l \frac{\left(\vec{v}_l - \vec{c}_k\right)\cdot\vec{n}_k}{\sqrt{2\vec{n}_k\cdot\vec{n}_l + 1}}.$$

The empirical value for alpha is

$$\alpha = \min\left(\left\|\mathbb{A}^{\top}\mathbf{I}\right\|^2, 1\right) = \min\left(\left[\sum_i w_{ij}\right]^2, 1\right)$$

where $\mathbb{A}$ is the operator that generates point fidelity proxy points and w_{ij} is as defined in Eq. 2 above.

Mesh refinement

The starting mesh we derive from density thresholding is relatively coarse (consisting of triangular cells that are large compared with organelle curvature). The initial mesh will typically also erroneously connect parts of the structure that should be separate (undersegmentation)—a desirable attribute as it is easier to separate areas that were erroneously joined than to join areas that were erroneously separated. As the optimization progresses, it will also expand or contract areas of the mesh unevenly, leading to a wide variation in mesh cell size. To resolve these issues, we interleave optimization steps (described above) with operations that manipulate the mesh, performing "remeshing" operations that lead to a more finely sampled mesh and ensure that the mesh is well formed for subsequent numerical operations (33), and "neck removal" operations, which cut the necks that form as the mesh wraps around areas that were erroneously joined in the starting mesh. These operations are performed every five iterations. Results are not strongly dependent on this frequency (see Fig. S3), although it should be regular enough to keep the mesh edge lengths fairly constant as parts of the mesh pull in. It is preferable to choose the total iteration number such that a few shrink-wrapping iterations are performed after the final remesh.

Remeshing (33) consists of a number of operations—splitting long edges, collapsing short edges, "flipping" edges where too many are incident on a vertex, and regularly spacing vertices along the surface. Together, these remeshing operations result in a well-formed mesh where the edge lengths are roughly constant, the number of edges incident on a single vertex is roughly constant, and the triangles are roughly equilateral. By adjusting the target edge length in the remeshing operation we can adjust the sampling of the mesh. Starting from the mean edge length of the starting mesh, we decrease the edge length linearly toward $\frac{\min_i \sigma_i}{2.5}$ or 0.4× the minimum localization precision—a value that lets us adequately sample the smallest resolvable features in our localization data set. Alternatively, a minimum edge length can be set by the user. This edge length should be sufficiently small to sample the curvature expected in the structure. In practice, a lower limit of 5 nm works well for the structures shown in this paper. The linear reduction of edge length during the course of optimization ensures that the iterative fitting makes large adjustments early in the fitting and fits detailed features later on.

To remove necks, vertices with unphysically high negative Gaussian curvature are deleted, and resulting holes in the mesh are stitched with fresh triangles. This operation removes false, thin necks in between portions of the surface. This operation is performed in regular intervals throughout our iterative fitting process.

Simulation and quality evaluation

To evaluate the fidelity and accuracy of our method, and to compare with the results obtained using SPR, we applied both methods to simulated data for which the ground truth surface was known. SMLM point clouds were simulated from a theoretical figure-eight, defined by a signed distance function (see supporting material). Simulations varied point cloud density and number of background localizations. We did not explicitly vary localization precision as, being mathematically equivalent to simple scaling of the structure, this should effect both methods equally.

Surfaces were fit to simulated point clouds and then compared with the theoretical structure giving rise to these point clouds. When making this comparison we must consider two types of error: 1) the distance from the true surface to the reconstructed surface and 2) the distance from the reconstructed surface to the true surface. Although these two metrics might seem redundant at first glance, they are not. It is possible for a surface to appear good under metric 1 while having bad performance under metric 2. An example of this is a structure that mostly closely follows the true surface, but also has blebs or extrusions away from the true surface. Because the distance in metric 1 just considers the parts of the reconstruction that are closest to the true structure, blebs and extrusions are not penalized and metric 1 returns a small distance. Similarly, a reconstruction that follows part of the ground truth correctly, but is truncated such that it does not extend into all areas of the ground truth, can score well on metric 2. A good reconstruction minimizes both of these metrics.

The distance between surfaces was calculated numerically as follows; a set of noise-free verification points were simulated exactly on the surface of the theoretical structure, and a set of noise-free points were simulated on the fit surface. The mean-square distance from the verification point set to its nearest neighbors in the mesh point set was computed as quality metric Q_1. The mean-square distance from the mesh point set to its nearest neighbors in the verification point set was computed as quality metric Q_2. Mesh quality was scored as a combination of the two error types:

$$Q = \sqrt{\frac{Q_1 + Q_2}{2}}$$

where Q is the root mean-square error, representing the average distance from the mesh to the theoretical structure.

Cell culture and sample preparation

U-2 OS cells (HTB-96; ATCC, Manassas, VA, USA, lot: 70008732) were grown in McCoy's 5A medium (Gibco, Waltham. MA, USA 16600-082) supplemented with 10% FBS (Gibco, 10438-026). These cells were subcultured with 0.05% Trypsin (Gibco).

Immunofluorescence samples were generally prepared as follows. Approximately 1 million cells in 90 μL were transfected via electroporation with about 10 μg of plasmid with a Super Electroporator NEPA21 Type II

(Nepa Gene, Chiba, Japan). The cells were then seeded onto coverslips that were, unless otherwise noted, treated in an ozone chamber for 30 min. Cells were chemically fixed the following day with 3% paraformaldehyde (Electron Microscopy Sciences, Hatfield, PA, USA 15710) and 0.1% glutaraldehyde (Electron Microscopy Sciences, 16019) for 15 min at room temperature while gently rocking. The glutaraldehyde fixation was quenched by washing the samples with 0.1% sodium borohydride in 1× PBS for 7 min followed by washing with 100 mM glycine in 1× PBS for 10 min. Samples were then rinsed three times with 1× PBS followed by a 3-min incubation at room temperature with permeabilization buffer (0.3% IGEPAL CA-630 + 0.05% Triton X-100 + 0.1% [w/v] BSA in 1× PBS) and another three rinses with 1× PBS. Samples were blocked for 1 h at room temperature in block buffer (0.05% IGEPAL CA-630 + 0.05% Triton X-100 + 5% normal goat serum in 1× PBS), followed by an overnight incubation with the primary antibody diluted in block buffer at 4°C while gently rocking. The following day, the samples were washed three times, for 5 min each wash, with wash buffer (0.05% IGEPAL CA-630 + 0.05% Triton X-100 + 0.2% [w/v] BSA in 1× PBS), incubated with secondary antibodies diluted in block buffer for 1 h at room temperature while gently rocking, washed three more times, for 5 min each wash, with wash buffer, and finally rinsed three times with 1× PBS. For astigmatic/4Pi-DNA-PAINT imaging of overexpressed mCherry-Sec61β and 4Pi-DNA-PAINT imaging of TOMM20-mCherry, samples were immunolabeled with rabbit anti-mCherry primary (Abcam, Cambridge, UK, ab167453) diluted 1:500 in block buffer and rabbit anti-TOMM20 primary (Santa Cruz Biotechnology, Dallas, TX, USA, sc-11415) diluted 1:500 in block buffer, respectively. Both samples were then labeled with an oligonucleotide-conjugated goat anti-rabbit IgG secondary antibody (Jackson ImmunoResearch, West Grove, PA, USA, 115-005-146) diluted 1:200 in block buffer, as described previously (34). For two-color 4Pi-STORM imaging of overexpressed mCherry-Sec61β and endogenously expressed TOMM20, samples were labeled with a combo of two mouse anti-mCherry primaries (GeneTex, Irvine, CA, USA, GTX630195 and GTX630189) each diluted 1:250 in block buffer and rabbit anti-TOMM20 primary (see above) diluted 1:500 in block buffer. They were then labeled with a goat anti-rabbit secondary conjugated to AF647 (Biotium, Fremont, CA, USA, 20812) diluted 1:1000 in block buffer and a goat anti-mouse secondary (Invitrogen, Waltham, MA, USA, A21245) with a single conjugated CF660C dye on each secondary antibody diluted 1:1000 in block buffer. The mCherry-Sec61β plasmid was acquired from Addgene (Watertown, MA, USA, 49155).

Microscopy

4Pi-SMS two-color samples of TOMM20 and mCherry-Sec61β were prepared and imaged using ratiometric dSTORM as described previously (35). 4Pi-SMS one-color DNA-PAINT samples were imaged on the same custom microscope, but with fluorogenic DNA-PAINT (see below). Astigmatic data were collected using a custom-built microscope described previously (36) with the only filter used being a bandpass filter (Semrock, Rochester, NY, USA, FF01-694/SP). Astigmatism was implemented by adding a cylindrical lens to the fluorescence light path.

All DNA-PAINT data were collected using the fluorogenic DNA-PAINT method described previously (34). In brief, samples were imaged using the following imager probe containing a dye and a quencher: 5'-Cy3B-AAGAAG-TAAAGGGAG-BHQ2-3'. This imager probe was diluted to 10 nM for both astigmatic and 4Pi imaging of mCherry-Sec61β and 1 nM for 4Pi-SMS imaging of TOMM20-mCherry in a high ionic-strength PBS-based buffer (1× PBS, 500 mM NaCl, 20 mM Na_2SO_3, and 1 mM Trolox [pH 7.3–7.5]).

RESULTS AND DISCUSSION

Validation on simulated SMLM data sets

A 3D figure-eight (two toruses touching) was simulated across a range of localization density and background values chosen to reflect the ranges we see in typical experiments (see supporting material). As both SPR and NanoWrap have a number of user-settable hyperparameters, a fair comparison of the methods requires a systematic search of the parameter space to avoid the possibility that we simply choose a disadvantageous SPR parameterization. For each condition, we therefore performed a grid search of the feasible parameter space for both SPR and NanoWrap. The resulting meshes were scored as described in quality evaluation. For each condition, the mesh with the lowest root mean-square error (Q, see simulation and quality evaluation) from each method was selected for comparison. This eliminates the possibility of user bias in parameter selection. The results are shown in Fig. 2.

Both NanoWrap and SPR work well when there is a high density of points and low background, and are capable of delivering a root mean-square error between ground truth and fitted surfaces, which is less than the localization precision ($\boldsymbol{\sigma}$). The combination of low background and high density is rare, however, in experimental data. As density decreases and background increases, SPR has a hard time fitting an accurate surface. In contrast, NanoWrap yields surface errors that are better than the localization precision ($\boldsymbol{\sigma}$) and can accurately estimate curvatures over a wide range of densities and background. Performance also degrades only slowly outside this space, with acceptable performance even at the very low localization densities that might be seen in live-cell SMLM.

To investigate the accuracy of our curvature estimates, we simulated a tapered cylinder varying in radius from 30 to 200 nm over a 2000-nm length (Figs. S4 and S5). As might be expected, the accuracy of curvature estimation depends on both localization density and precision, becoming unreliable at very low densities and poor localization precision. Based on these simulations, we are likely to underestimate curvature when the true curvature radius is less than approximately three times the localization precision or about twice the median distance between localizations.

The sensitivity of our algorithm to parameter choice is discussed in detail in the supporting material (Note S3 and Fig. S6). In brief, curvature weight is the most important user-settable parameter and is used to balance the point fidelity and curvature terms. The optimal value of this parameter increases as point density and background increase. The corresponding minimum in surface error, however, is broad, and small changes in the parameter value do not have large effects on surface quality.

Application to experimental SMLM data

We initially tested our NanoWrap algorithm on data acquired using 4Pi-SMS microscopy (35,37) as this provides high-precision isotropic localization sufficient to capture the finest details of small intracellular organelles such as the ER. When applied to these data, NanoWrap results in

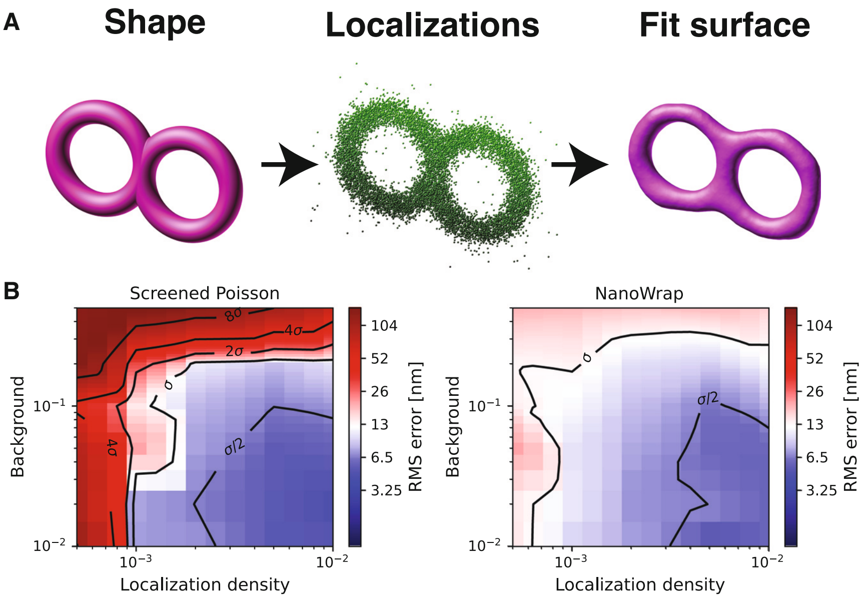

FIGURE 2 A comparison of SPR and NanoWrap on simulated point clouds of a 3D figure-eight, which is approximately 800 nm in diameter and 100 nm thick. (*A*) Our simulation method of going from a signed distance function to a set of localizations to a fit surface. (*B*) Heatmap plots of screened Poisson reconstruction and NanoWrap as functions of simulated localization density and background. For reference, the experimental SMLM data shown in Figs. 3 and 4 had a median localization density of approximately 6×10^{-2}nm^{-2} and background ration of 2×10^{-1}. To see this figure in color, go online.

an approximation of the ER surface (Fig. 3), which is consistent with both the observed Sec61β localizations and our knowledge of ER structure. Cross sections of the data allow us to visually confirm that the surface approximation is well matched to the localization point cloud data (Fig. 3, *E–G*). Quantification of ER tubule diameter from the surface reconstruction is in good agreement with previously reported values (38) (see Fig. S9).

Although advanced localization techniques such as the combination of 4Pi-SMS and DNA-PAINT (or, alternatively, MIN-FLUX (39)) are likely to yield the highest-quality reconstructions, these techniques are not as widely available as more accessible methods such as astigmatic 3D DNA-PAINT. To demonstrate that the algorithm is not restricted to the ER and can be used across a broader range of instrumentation and labeling approaches, surfaces were additionally generated for mitochondria and using 4Pi two-color dSTORM as well as astigmatic 3D DNA-PAINT, with the results shown in Fig. 4. Notably, a good reconstruction was still possible from astigmatic data despite the poorer axial localization precision compared with 4Pi-SMS (Fig. 4, *G–I*). This can be attributed to the fact that our surface-fitting approach takes this anisotropic error into account when weighting the attraction force and finds the average surface that passes through multiple nearby localizations (even if the scatter of points in z is larger, poorer axial precision will not change their mean position).

To achieve accurate curvature estimates using NanoWrap, localization precision must be smaller than the radius of curvature of the feature of interest (Fig. S5). Given sufficient density and localization precision, NanoWrap is able to follow this curvature profile of a tapered cylinder smoothly (Fig. S4). At too low a density, the curvature is slightly underestimated. To reconstruct the ER (which has typical tubule diameters of around 100 nm) (38) we therefore recommend a median axial localization precision better than 30 nm (easily achievable using astigmatic 3D DNA-PAINT, but challenging for non-4Pi dSTORM), but predict that acceptable results will be able to be obtained on larger structures (e.g., mitochondria with a diameter of 0.5–1 μm) for all standard 3D localization microscopy modalities. As implemented, NanoWrap will generally recover holes bigger than around three times the localization precision, but will occasionally miss small holes or fenestrations in sheet like structures that might be discernible when examining the localization cloud by eye (see also Note S3 and Fig. S8). This is both because the reliable detection of small holes is a surprisingly tricky computational problem and because of a deliberate design choice to bias the starting surface this way so as to have a predictable failure mode and to limit the types of topological errors that must be handled in subsequent steps. Attempting to be bias free leads to fragmentation of structures and the generation of spurious holes before all true holes are reliably detected. Although not worse than other available algorithms, improving the topological accuracy of NanoWrap is a promising target for future research.

The surface approximations we create allow exciting new opportunities for quantitative analysis, permitting, for example, the measurement of curvature at each point on the surface (Fig. 3, *A* and *D*). In two-color data, each color channel can be fit independently (Fig. 4, *D–F*) to yield relationships such as distance between surfaces to be calculated and visualized on the surfaces themselves (see Fig. S10). This particular use has the potential to provide information about the location of membrane contact sites between the ER and other organelles that exhibit exceptionally small distances, 10–30 nm, between membranes (40). Analogous approaches can be used to measure distances to individual localizations in a second channel allowing the study of proteins that are partially or fully cytosolic.

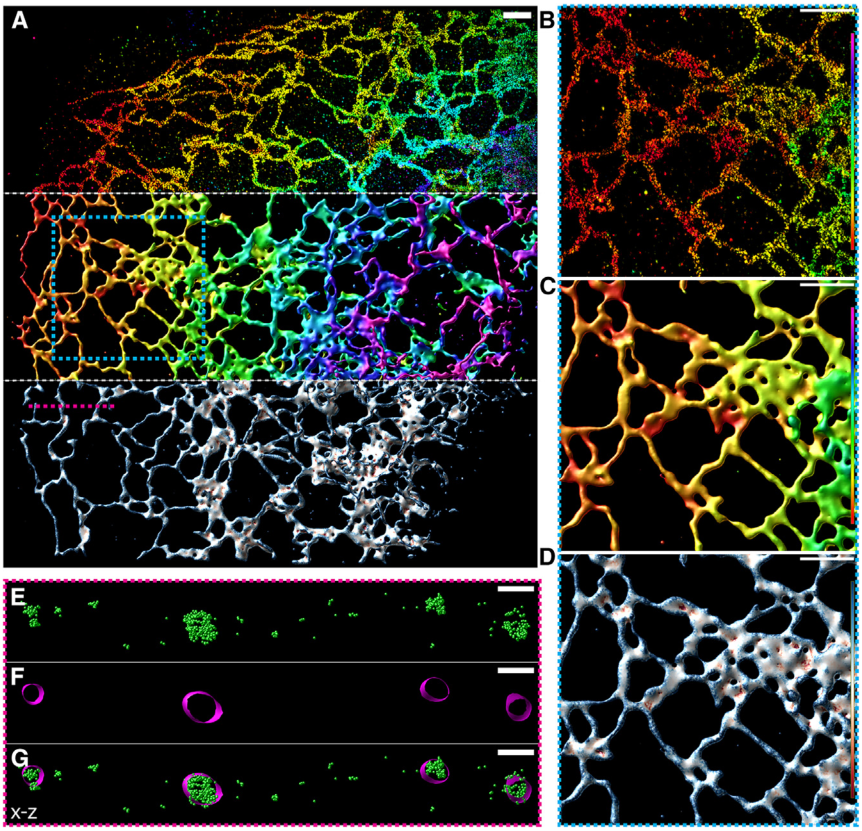

FIGURE 3 (*A*) 4Pi fluorogenic DNA-PAINT data of overexpressed mCherry-Sec61β, an endoplasmic reticulum membrane protein. Top: point cloud data displayed as 10 nm point sprites with alpha set to 0.5 and colored by each point's position in *z* according to the lookup table described in (*B* and *C*). Middle: NanoWrap surface created based on the point cloud data colored by each vertex's position in *z* according to the lookup table described in (*B* and *C*). Bottom: the same surface, but colored by the mean curvature at each vertex according to the lookup table described in (*D*). (*B–D*) ROI shown by the hashed cyan box in (*A*) displayed in the three different ways described in (*A*). (*B* and *C*) Lookup table (from *bottom* to *top*): 0 to 800 nm. (*D*) Lookup table: -0.01 to 0.01 nm^{-1}. (*E–G*) Cross section in *x-z* shown by the hashed magenta line in (*A*). (*E*) Point cloud displayed as 10-nm green spheres. (*F*) Surface displayed in magenta. (*G*) Point cloud and surface displayed together. Scale bars, 1 μm (*A–D*) and 200 nm (*E–G*). To see this figure in color, go online.

CONCLUSION

Examining the interplay of membrane surfaces and proteins is critical to understanding cellular function (41). The surface-fitting algorithm described in this paper provides a new way for researchers to segment and quantify membrane structure from single-molecule localization microscopy data, and to subsequently study membrane-membrane and membrane-protein interactions at biophysically relevant size scales. The algorithm and its resulting structures are biophysically informed: localizations are fit based on expected fluorophore blinking behavior and labeling inefficiencies, and the curvature smoothing is inspired by the Canham-Helfrich bending energy functional, an established model for biological membranes (32,42). This incorporation of previous information to achieve better reconstructions is a natural next step in biological membrane surface approximation and, more generally, to the analysis of noisy imaging data as a whole. It complements exciting new machine learning techniques for segmentation and analysis (11,43,44). In contrast with machine learning approaches, it does not require large volumes of manually annotated training data, and has a clear basis in biophysical theory.

The mathematics behind NanoWrap are not intrinsically limited to SMLM, and the ability to deal with noisy data, incomplete labeling, and anisotropic resolution should be a significant benefit for other high-resolution modalities (e.g., stimulated emission depletion and electron microscopy). We look forward to adapting the method to these domains. A particularly exciting potential application of our technique is to live-cell super-resolution, which is heavily constrained by the achievable localization density (SMLM) (45) or photon count (stimulated emission depletion) (46). The ability of NanoWrap to recover accurate organelle surfaces from very low localization densities (Fig. 2) suggests an approximately 10- to 20-fold reduction in the required localization density. When combined with multiemitter approaches, this should be enough to make high-quality live super-resolution imaging of dynamic organelles possible.

In summary, NanoWrap outperforms previously demonstrated methods of SMLM membrane estimation and enables high-fidelity membrane reconstructions across a wide range of localization densities and backgrounds. For ease-of-use, shareability and adaptability, it is packaged as open-source software and is accessible via an interactive GUI. It is sufficiently fast and memory efficient to be used on standard lab computers (e.g., midrange laptops). Nano Wrap is available for download as a PYME plugin at https://github.com/python-microscopy/ch-shrinkwrap.

SUPPORTING MATERIAL

Supporting material can be found online at https://doi.org/10.1016/j.bpj.2023.06.010.

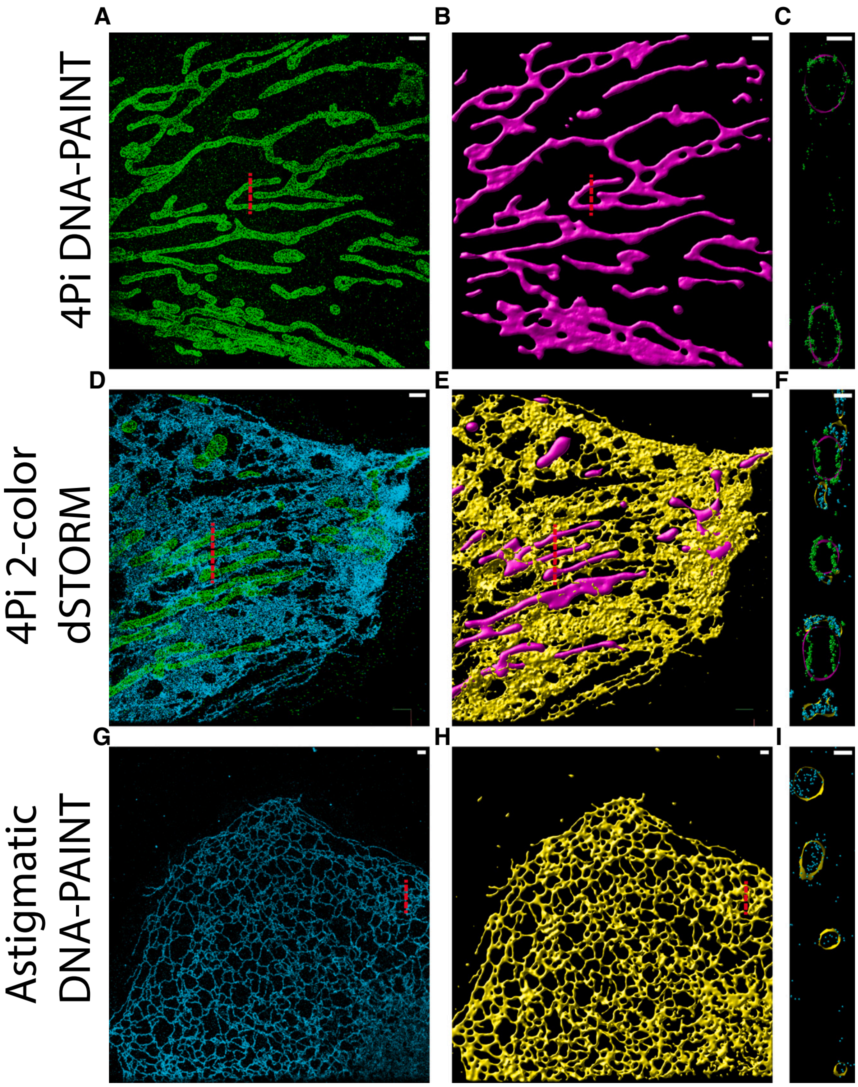

FIGURE 4 Application of NanoWrap on data from varying SMLM imaging modes. (*A–C*) 4Pi fluorogenic DNA-PAINT localizations from outer mitochondrial membrane protein TOMM20 (*green*) and their resulting surface (*magenta*). (*D–F*) Two-color 4Pi dSTORM localizations of TOMM20 (*green*) and overexpressed endoplasmic reticulum membrane protein Sec61β (*cyan*) and their resulting surfaces (*magenta* and *yellow*, respectively). (*G–I*) Astigmatic 3D fluorogenic DNA-PAINT localizations from overexpressed Sec61β (*cyan*) and their resulting surface (*yellow*). (*A*, *D*, and *G*) *x-y* view of localized point cloud. (*B*, *E*, and *H*) *x-y* view of resulting surface. (*C*, *F*, and *I*) *x-z* view of surfaces and points overlaid. Scale bars, 1 μm (*A*, *B*, *D*, *E*, *G*, and *H*) and 200 nm (*C*, *F*, and *I*). To see this figure in color, go online.

AUTHOR CONTRIBUTIONS

Z.M. and D.B. designed and implemented the algorithm. L.A.F. tested the algorithm, prepared samples, imaged the biological data, and created the surface reconstructions of the biological data. J.B. provided critical feedback on the algorithm and the biological test case. All authors wrote the manuscript.

ACKNOWLEDGMENTS

The authors thank Michael Murrell, Megan King, Andrew Barentine, Yongdeng Zhang, Lena Schroeder, Florian Schueder, and Frederic Pincet for helpful discussions. We acknowledge funding from the Wellcome Trust (203285/B/16/Z) and NIH (R01GM118486, T32GM007223, and T32EB019941). This work is solely the responsibility of the authors and does not necessarily represent the official views of the NIH.

DECLARATION OF INTERESTS

J.B. discloses a significant financial interest in Bruker Corp., Hamamatsu Photonics, and panluminate Inc.

SUPPORTING CITATIONS

References (47,48) appear in the supporting material.

REFERENCES

1. Quemeneur, F., J. K. Sigurdsson, …, D. Lacoste. 2014. Shape matters in protein mobility within membranes. *Proc. Natl. Acad. Sci. USA*. 111:5083–5087.

2. Westrate, L. M., J. E. Lee, …, G. K. Voeltz. 2015. Form Follows Function: The Importance of Endoplasmic Reticulum Shape. *Annu. Rev. Biochem.* 84:791–811.

3. Tarazón, E., E. Roselló-Lletí, …, M. Rivera. 2017. Changes in human Golgi apparatus reflect new left ventricular dimensions and function in dilated cardiomyopathy patients. *Eur. J. Heart Fail.* 19:280–282. https://doi.org/10.1002/ejhf.671.

4. Kaksonen, M., and A. Roux. 2018. Mechanisms of clathrin-mediated endocytosis. *Nat. Rev. Mol. Cell Biol.* 19:313–326. http://www.nature.com/articles/nrm.2017.132.

5. Haupt, A., and N. Minc. 2018. How cells sense their own shape – mechanisms to probe cell geometry and their implications in cellular organization and function. *J. Cell Sci.* 131:jcs214015.

6. Colina-Tenorio, L., P. Horten, …, H. Rampelt. 2020. Shaping the mitochondrial inner membrane in health and disease. *J. Intern. Med.* 287:645–664.

7. Dias, C., and J. Nylandsted. 2021. Plasma membrane integrity in health and disease: significance and therapeutic potential. *Cell Discov.* 7:4. http://www.nature.com/articles/s41421-020-00233-2.

8. Goyal, U., and C. Blackstone. 2013. Untangling the web: Mechanisms underlying ER network formation. *Biochim. Biophys. Acta.* 1833:2492–2498. https://linkinghub.elsevier.com/retrieve/pii/S0167488913001699.

9. Ladinsky, M. S., D. N. Mastronarde, …, L. A. Staehelin. 1999. Golgi Structure in Three Dimensions: Functional Insights from the Normal Rat Kidney Cell. *J. Cell Biol.* 144:1135–1149.

10. Hecksel, C. W., M. C. Darrow, …, W. Chiu. 2016. Quantifying variability of manual annotation in cryo-electron tomograms. *Microsc. Microanal.* 22:487–496.

11. Heinrich, L., D. Bennett, …, D. Nguyen. 2021. Whole-cell organelle segmentation in volume electron microscopy. *Nature.* 599:141–146.

12. Dahlberg, P. D., and W. E. Moerner. 2021. Cryogenic Super-Resolution Fluorescence and Electron Microscopy Correlated at the Nanoscale. *Annu. Rev. Phys. Chem.* 72:253–278.

13. Griffiths, G., and J. M. Lucocq. 2014. Antibodies for immunolabeling by light and electron microscopy: not for the faint hearted. *Histochem. Cell Biol.* 142:347–360.

14. Bond, C., A. N. Santiago-Ruiz, …, M. Lakadamyali. 2022. Technological advances in super-resolution microscopy to study cellular processes. *Mol. Cell.* 82:315–332.

15. Milo, R., and R. Phillips. 2016. Cell Biology by the Numbers. Taylor & Francis Group.

16. Wang, Z., and M. Menenti. 2021. Challenges and Opportunities in Lidar Remote Sensing. *Front. Remote Sens.* 2. https://doi.org/10.3389/frsen.2021.641723.

17. Curless, B. 1999. From Range Scans to 3D Models. *SIGGRAPH Comput. Graph.* 33:38–41. https://doi.org/10.1145/345370.345399.

18. Kazhdan, M., and H. Hoppe. 2013. Screened poisson surface reconstruction. *ACM Trans. Graph.* 32:1–13.

19. Yoon, J., C. J. Comerci, …, W. E. Moerner. 2019. Revealing Nanoscale Morphology of the Primary Cilium Using Super-Resolution Fluorescence Microscopy. *Biophys. J.* 116:319–329.

20. Lelek, M., M. T. Gyparaki, …, C. Zimmer. 2021. Single-molecule localization microscopy. *Nat. Rev. Methods Primers.* 1:39.

21. Koo, B. K., Y. K. Choi, …, B. T. Choi. 2005. Shrink-Wrapped Boundary Face Algorithm for Mesh Reconstruction from Unorganized Points. *ETRI J.* 27:235–238. https://doi.org/10.4218/etrij.05.0204.0027.

22. Alliez, P., D. Cohen-Steiner, …, M. Rouxel-Labbé. 2022. 3D Alpha Wrapping. *In* CGAL User and Reference Manual CGAL Editorial Board https://doc.cgal.org/5.5/Manual/packages.html#PkgAlphaWrap3.

23. Hanocka, R., G. Metzer, …, D. Cohen-Or. 2020. Point2Mesh: A Self-Prior for Deformable Meshes. Preprint at arXiv. https://doi.org/10.48550/arXiv.2005.11084.

24. Zhao, Y., S. M. Schreiner, …, S. G. J. Mochrie. 2016. Improved Determination of Subnuclear Position Enabled by Three-Dimensional Membrane Reconstruction. *Biophys. J.* 111:19–24. https://doi.org/10.1016/j.bpj.2016.05.036.

25. Brakke, K. A. 1992. The surface evolver. *Exp. Math.* 1:141–165.

26. Terasaki, M., T. Shemesh, …, M. M. Kozlov. 2013. Stacked Endoplasmic Reticulum Sheets Are Connected by Helicoidal Membrane Motifs. *Cell.* 154:285–296.

27. Marin, Z., M. Graff, …, D. Baddeley. 2021. PYMEVisualize: an open-source tool for exploring 3D super-resolution data. *Nat. Methods.* 18:582–584.

28. Meagher, D. 1980. Octree Encoding: A New Technique for the Representation, Manipulation and Display of Arbitrary 3-D Objects by Computer. *In* Technical Report Technical Report IPL-TR-80-111 Rensselaer Polytechnic Institute.

29. Baddeley, D., M. B. Cannell, and C. Soeller. 2010. Visualization of localization microscopy data. *Microsc. Microanal.* 16:64–72.

30. Schaefer, S., and J. Warren. 2005. Dual marching cubes: Primal contouring of dual grids. *Comput. Graph. Forum.* 24:195–201.

31. Press, W. H., S. A. Teukolsky, …, B. P. Flannery. 2007. Numerical Recipes 3rd Edition. *In* The Art of Scientific Computing Cambridge University Press.

32. Nelson, D., T. Piran, and S. Weinberg. 2004. Statistical Mechanics of Membranes and Surfaces. World Scientific.

33. Botsch, M., and L. Kobbelt. 2004. A remeshing approach to multiresolution modeling. *ACM International Conference Proceeding Series.* 71:185–192.

34. Chung, K. K. H., Z. Zhang, …, J. Bewersdorf. 2022. Fluorogenic DNA-PAINT for faster, low-background super-resolution imaging. *Nat. Methods.* 19:554–559.

35. Zhang, Y., L. K. Schroeder, …, J. Bewersdorf. 2020. Nanoscale subcellular architecture revealed by multicolor three-dimensional salvaged fluorescence imaging. *Nat. Methods.* 17:225–231.

36. Tyson, J., K. Hu, …, A. Schepartz. 2021. Extremely Bright, Near-IR Emitting Spontaneously Blinking Fluorophores Enable Ratiometric Multicolor Nanoscopy in Live Cells. *ACS Cent. Sci.* 7:1419–1426.

37. Huang, F., G. Sirinakis, …, J. Bewersdorf. 2016. Ultra-High Resolution 3D Imaging of Whole Cells. *Cell.* 166:1028–1040. https://doi.org/10.1016/j.cell.2016.06.016.

38. Schroeder, L. K., A. E. S. Barentine, …, S. Bahmanyar. 2019. Dynamic nanoscale morphology of the ER surveyed by STED microscopy. *J. Cell Biol.* 218:83–96. https://doi.org/10.1083/jcb.201809107.

39. Gwosch, K. C., J. K. Pape, …, S. W. Hell. 2020. MINFLUX nanoscopy delivers 3D multicolor nanometer resolution in cells. *Nat. Methods.* 17:217–224.

40. Wu, H., P. Carvalho, and G. K. Voeltz. 2018. Here, there, and everywhere: The importance of ER membrane contact sites. *Science.* 361, eaan5835.

41. Bassereau, P., R. Jin, …, T. R. Weikl. 2018. The 2018 biomembrane curvature and remodeling roadmap. *J. Phys. D Appl. Phys.* 51, 343001. https://doi.org/10.1088/1361-6463/aacb98.

42. Helfrich, W. 1973. Elastic Properties of Lipid Bilayers: Theory and Possible Experiments. *Z. Naturforsch. C.* 28:693–703.

43. Liu, Z., L. Jin, …, Y. Xu. 2021. A survey on applications of deep learning in microscopy image analysis. *Comput. Biol. Med.* 134, 104523.

44. Melanthota, S. K., D. Gopal, …, N. Mazumder. 2022. Deep learning-based image processing in optical microscopy. *Biophys. Rev.* 14:463–481. https://doi.org/10.1007/s12551-022-00949-3.

45. Baddeley, D., and J. Bewersdorf. 2018. Biological Insight from Super-Resolution Microscopy: What We Can Learn from Localization-Based Images. *Annu. Rev. Biochem.* 87:965–989. https://doi.org/10.1146/annurev-biochem-060815-014801.

46. Vicidomini, G., P. Bianchini, and A. Diaspro. 2018. STED super-resolved microscopy. *Nat. Methods.* 15:173–182.

47. McNeel, R. 2010. Rhinoceros 3D, Version 8.0. Robert McNeel & Associates, Seattle, WA.

48. Cignoni, P., M. Callieri, …, G. Ranzuglia. 2008. MeshLab: an Open-Source Mesh Processing Tool. *In* Eurographics Italian Chapter Conference. V. Scarano, R. D. Chiara, and U. Erra, eds The Eurographics Association.

Biophysical Journal, Volume *122*

Supplemental information

Extracting nanoscale membrane morphology from single-molecule localizations

Zach Marin, Lukas A. Fuentes, Joerg Bewersdorf, and David Baddeley

SUPPLEMENTARY MATERIAL

S.1 Point cloud simulation

In order to demonstrate the effectiveness of the algorithm, it was necessary to generate biologically-motivated test structures. To do this, we established a constructive solid geometry library where each primitive is represented as a signed distance function (SDF). An SDF describes an object by taking a point location as input and returning the signed distance between the point and the surface of the object. An example SDF is that of a sphere of radius $R \in \mathbb{R}$, $d(p) = ||p|| - R$, where $p \in \mathbb{R}^3$. $d(p) = 0$ indicates that point p is on the surface of the sphere. Points located within the sphere have a negative distance to the sphere surface and points outside of the sphere have a positive distance to the sphere surface.

Points were generated from a test structure using Monte-Carlo sampling of an octree of the structure's SDF. The octree subdivides space that straddles where the SDF goes to zero until a fixed sampling rate dx is reached. The center of each octree box with an absolute SDF value of less than dx is kept with a probability of p and rejected otherwise. This results in a point set of size S.

Next, non-specific localizations are simulated. For a given noise fraction n, $J = \frac{nS}{1-n}$ additional points are generated with uniform randomness over the bounding box of the point set. This ensures $J/T = n$ for $T = J + S$ total points.

To simulate localization precision, each point i in the resulting point set is jittered by sampling values from a Gaussian with mean μ_i equivalent to the location of point $i \in 0..T-1$ and standard deviation $\boldsymbol{\sigma}_{i,e}$ for $e \in x, y, z$. Up to N samples are generated per point. The total point set is then clipped to size T via random uniform selection of points.

The uncertainty $\boldsymbol{\sigma}_{i,e}$ is calculated by simulating an exponential distribution with a mean value of ρ, corresponding to the average photon count of a simulated localization. This distribution is clipped to only contain values greater than a background photon count b, simulating the noise floor for data collection. For the remaining counts, the first S are selected and

$$\sigma_{i,e} = \frac{r_e}{2.355\sqrt{\rho_i}}$$

where r_e is the resolution of the simulated system along axis e.

S.2 NanoWrap parameters

To generate the parameter space for evaluation of SPR meshes derived from simulated data in Figure 2, we followed recommendations in (18). For SPR we estimated normals from 10, 30, 50 and 100 nearest neighbors, set the smoothing parameter $\alpha = 0, 1, 2$ and 4, and set the samples-per-node to 1.5, 5, 10, 20 and 30. We used an octree depth of 8, 8 Gauss-Seidel relaxations, and a scale factor of 1.1.

For our method, we compared maximum number of iterations 19 and 39, a variety of starting threshold densities, 5×10^{-6}, 1×10^{-5}, 2×10^{-5}, 5×10^{-5}, 1×10^{-4}, 2×10^{-4}, 4×10^{-4}, 1×10^{-3}, 2×10^{-3}, and $\lambda = 10, 15, 25$. We remeshed every 5 iterations.

	Max iters	Curvature weight	Remesh frequency	Neck first iter	Punch frequency	Kc	Minimum edge length	Smooth curvature	Truncate at
Figure 3	19	15	5	0	0	1.0	5.0	True	1000
Figure 4 A-C	39	10	5	0	0	1.0	5.0	True	1000
Figure 4 D-F (ER)	19	10	5	0	0	1.0	5.0	True	1000
Figure 4 D-F (Mito)	69	30	5	0	0	1.0	5.0	True	1000
Figure 4 G-I	15	10	5	0	0	1.0	5.0	True	1000

Table S1: Parameters used for iterative surface fitting of experimental data in this paper.

S.3 Parameter sensitivity

The search of the parameter space used to quantify RMS surface error (Figure 2) also permits an analysis of the sensitivity of that error to parameter choices. As the starting surface is an isosurface on density, the optimal choice of the threshold parameter for this is expected to correlate to labeling density. In practice, however, as long as the starting estimate is not too far off, the subsequent optimisation steps will ensure good surface quality. Because the number of localizations pulling on a mesh face

also correlates with density, the curvature weight required to balance the point attraction force is also expected to increase with increasing density. Figure S6 shows this is the case, although the minimum in RMS error is fairly broad indicating that the algorithm is not overly sensitive to the exact curvature weight. Increasing the background at a constant density shifts the optimal curvature weight to slightly higher values, but is not as significant as a change in density. The underlying curvature of the structure itself has only a very slight effect on the optimal curvature weight (Figure S7) the localization errors are already considered in the algorithm we do not expect a strong change in the optimal curvature weight with changing localization precision.

Unlike the RMS surface error, which can tolerate quite large variations in the starting surface, estimating the correct genus (number of holes) depends strongly on the threshold used to generate the starting surface. This is consistent with other shrink-wrapping approaches, which rely on manual thresholds to set hole size (21, 22). We attempt to address this automatically in NanoWrap, but to avoid obvious misrepresentation of underlying structure, the topology modification routines used during NanoWrap's shrink-wrapping iterations are quite conservative about when they will change the topology - i.e. they will only change topology if it is very obvious the current topology is wrong. They are also more reliable at removing spurious connections and holes than adding missing connections or holes. Because we bias the starting surface to be slightly outside the structure of interest, we are more likely to miss holes in areas of low density than to generate spurious holes. To assess the ability of our method to accurately capture membrane topology, we performed simulation of a synthetic structure with a range of hole sizes (Supplementary Figure S8). To avoid gaming the analysis we used an automated algorithm to set the threshold for the starting surface (see below). We also chose the curvature weight so as to minimise RMS error, not hole count. Under these conditions, the ability to capture a hole depends on a combination of localisation density, precision, and background level. At high densities and low levels of background, we can typically capture holes that are larger than around 3 times the localization precision, although we occasionally miss a hole we really should have captured. Manual tweaking of the parameters could have achieved improved the results. Correctly estimating surface genus is an area where we believe there is scope for improvement in our algorithm, especially in deciding when to modify the topology as we iterate.

Automatic threshold selection for starting surfaces

We expect that the starting surface should be close to the localizations used to generate it, and the median distance from each localization to the starting surface can be used as quality metric for the surface. By trying different thresholds within an optimisation routine (we use bisection) a threshold value leading to a good starting surface can be chosen. It practice it is helpful to aim for a distance (e.g. -10 to -40 nm) which puts the localizations just inside the surface and avoids the generation of spurious holes. This algorithm can be activated by selecting the 'Auto threshold' box in the Dual Marching Cubes settings, and will almost always generate good starting surfaces. Manual tweaking can, however, be useful if there are topological features (e.g. holes) which are right at the limit of being resolvable with the given localisation precision and densities.

Rational choice of curvature weighting

Similar to choosing a threshold for the starting surface, the distance between localizations and the reconstructed membrane can be a useful metric to determine whether an appropriate curvature weight has been selected. This time, however, we look at the distribution of distances rather than their median. Localizations are expected to scatter about the true membrane position due to their localisation error, with the distance between a localisation and the true position of that molecule expected to follow a scaled Chi(3) distribution. The choice of curvature weight is appropriate if the distribution of distances between localizations and the membrane is similar to that predicted by the localisation error. If the spread is larger, the curvature weight is too high, but if the spread is less than that predicted by localization error the algorithm is over-fitting to the points and the curvature weight should be increased. For performance reasons, we have not automated this, and setting the curvature weight remains a manual process. A function to plot the distance distributions is available under *Mesh->Show shrinkwrap residuals* within PYMEVis.

These distributions are also useful for showing if the number of iterations is suitable - if the residual distribution is heavily weighted to negative distances (inside the surface) this indicates that either more iterations are likely needed to allow the surface to converge.

Supplementary Figures

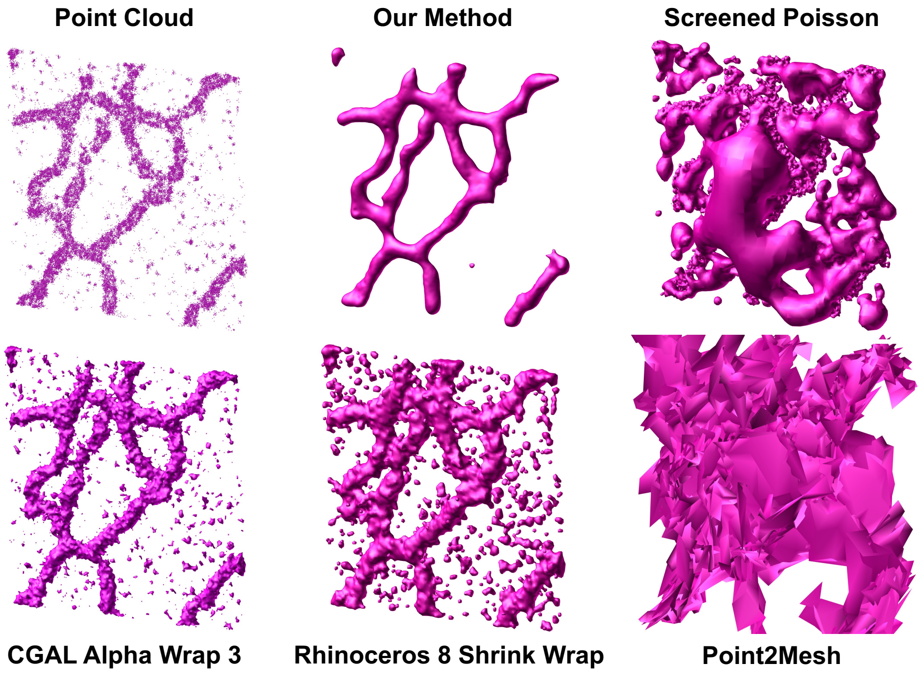

Figure S1: A comparison of our method to other common methods used to generate surfaces from a point cloud. Top left is an example point cloud from a subregion of an endoplasmic reticulum. A surface is generated from this point cloud with NanoWrap, Screened Poisson Reconstruction (18), CGAL's Alpha Wrap (22), Rhinoceros 8's shrink wrap algorithm (47) and Point2Mesh (23). NanoWrap allows the surface to adapt per-point based on localization positions and achieves a better representation of the underlying structure than other methods, which strictly adhere to the point cloud or generate a surface at a constant offset from each point. We manually experimented with the input hyperparameters of each algorithm to achieve the best surface in each case. A grid search may yield better surfaces for each of these methods, but we do not expect significant improvement, due to the nature of the input data. Both SPR and Point2Mesh require point normals as input. Point normals were estimated using Meshlab's (48) estimator. Note that these methods may perform well in the presence of true normals for a point cloud, but normal estimation usually relies on neighboring points, including noise points that confound the estimate. Point2Mesh requires the user to provide an initial starting surface. We provided it the same starting surface we used for our approach, which is a dual marching cubes (30) approximation on the density of the point cloud.

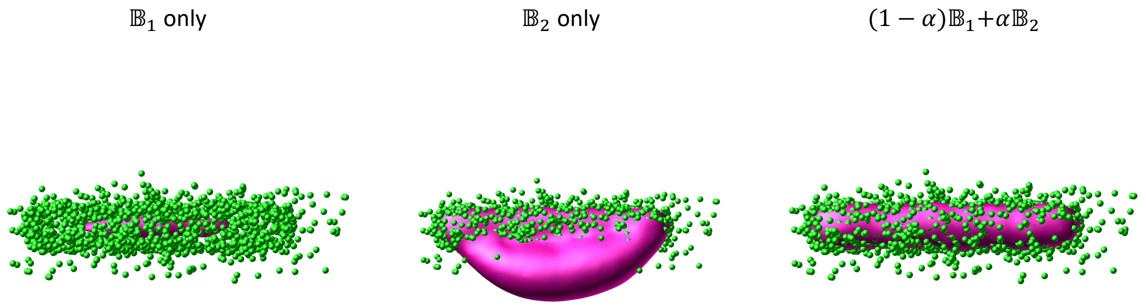

Figure S2: This figure demonstrates the independent influence of curvature forces $\mathbb{B}_1$ and $\mathbb{B}_2$ on the final NanoWrap surface. The localization point cloud is shown in green and the surface fitting these points is shown in magenta. $\mathbb{B}_1$ has area-minimizing tendencies and the surface pulls through the point cloud. $\mathbb{B}_2$'s preservation of area means that areas of the mesh far from point influence can establish stable "blebs" protruding from the true structure. The combination of forces subject to a point influence weighting α creates an accurate reconstruction of a surface. See the methods section for more details.

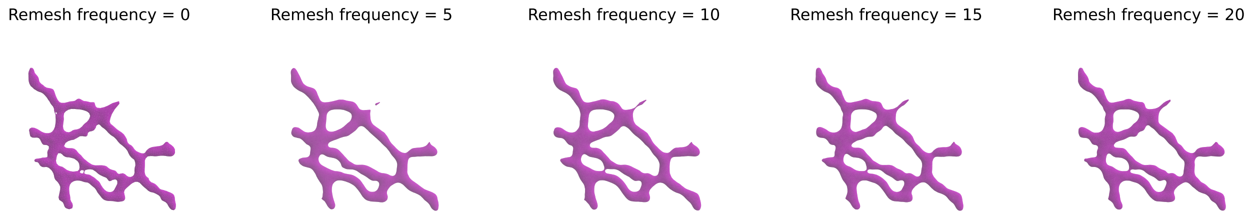

Figure S3: A NanoWrap surface fit over 39 iterations, remeshed every 0, 5, 10, 15, 20 iterations. The structure does not change significantly depending on remesh frequency.

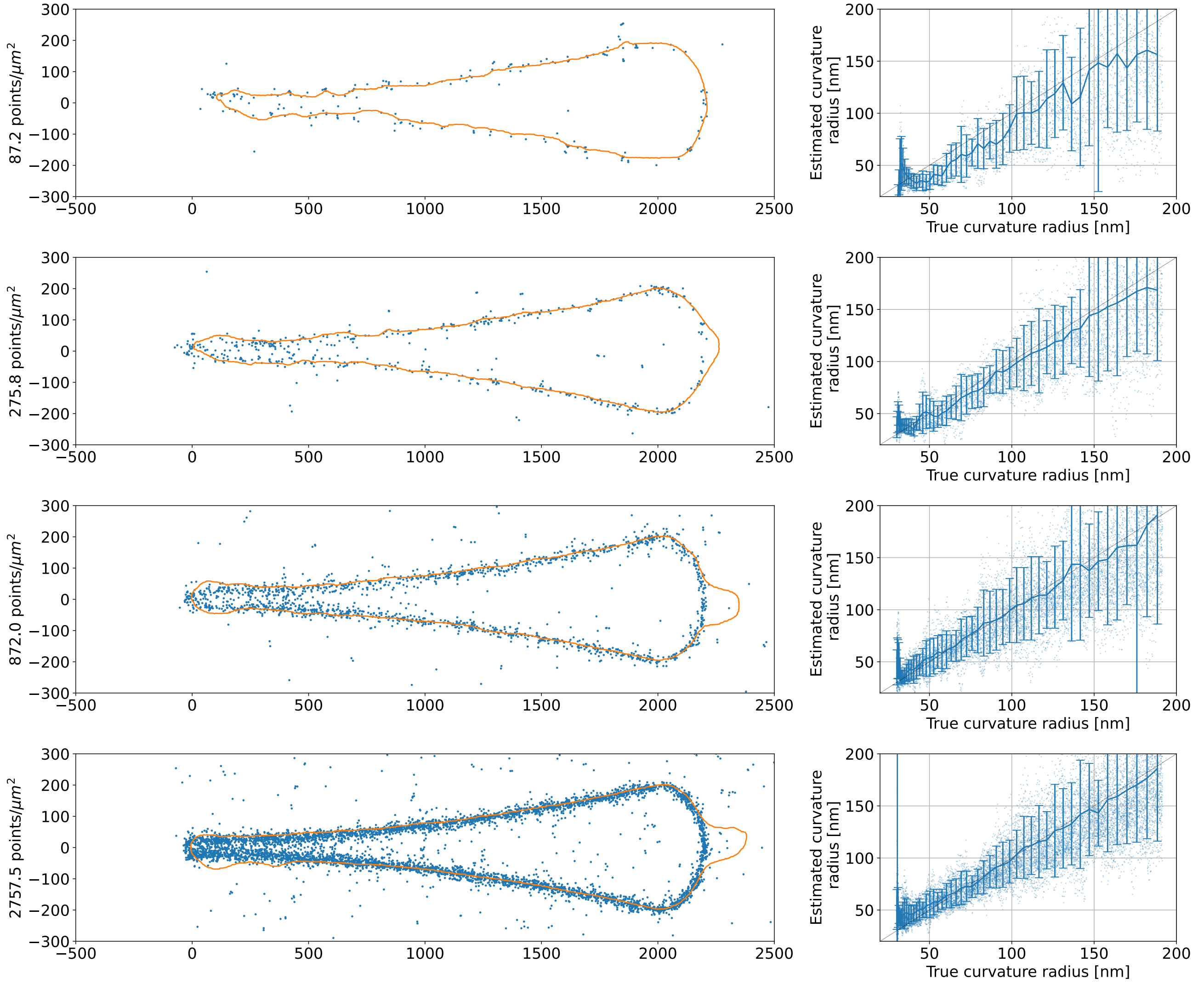

Figure S4: The radius of curvature of a reconstructed surface of a tapered cylinder as a function of density. The tapered cylinder's diameter scales between 30 and 200 nm over a 2000 nm distance as the square of the distance along the cylinder. The left column shows a cross section of the fit point cloud (blue) and the reconstructed surface (orange). The right column shows the estimated radius of curvature for each vertex of the fit surface along with an error bar indicating the full range of curvature estimates extracted from the vertices for each expected radius of curvature. As the density increases, the minimum radius of curvature that can be estimated decreases. The point cloud localization precision is fixed at 11 nm.

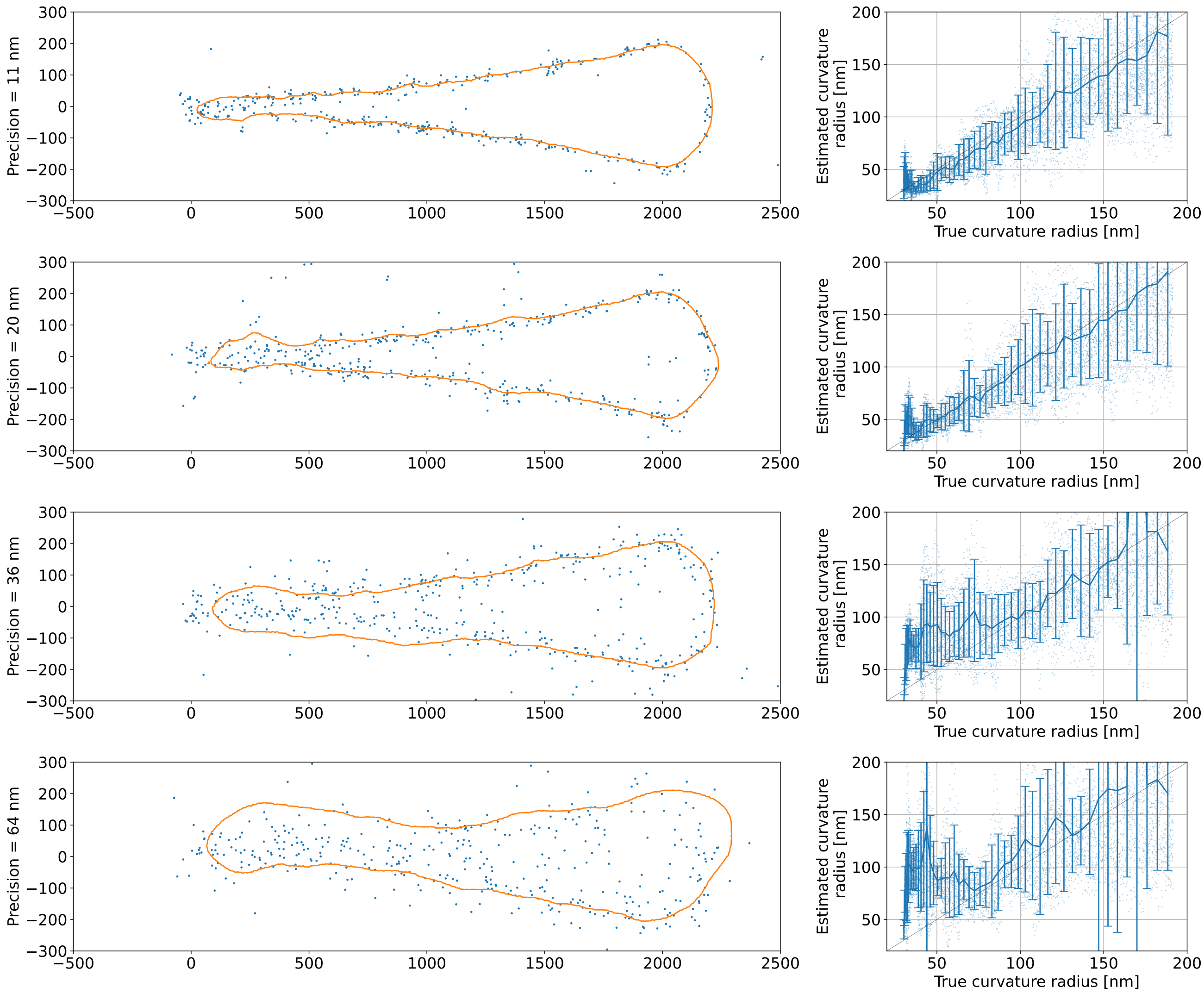

Figure S5: The radius of curvature of a reconstructed surface of a tapered cylinder as a function of localization precision. The tapered cylinder's diameter scales between 30 and 200 nm over a 2000 nm distance as the square of the distance along the cylinder. The left column shows a cross section of the fit point cloud (blue) and the reconstructed surface (orange). The right column shows the estimated radius of curvature for each vertex of the fit surface along with an error bar indicating the full range of curvature estimates extracted from the vertices for each expected radius of curvature. As the precision increases, the minimum radius of curvature that can be estimated scales to match. The point cloud density is fixed at 218 points μm^{-2}.

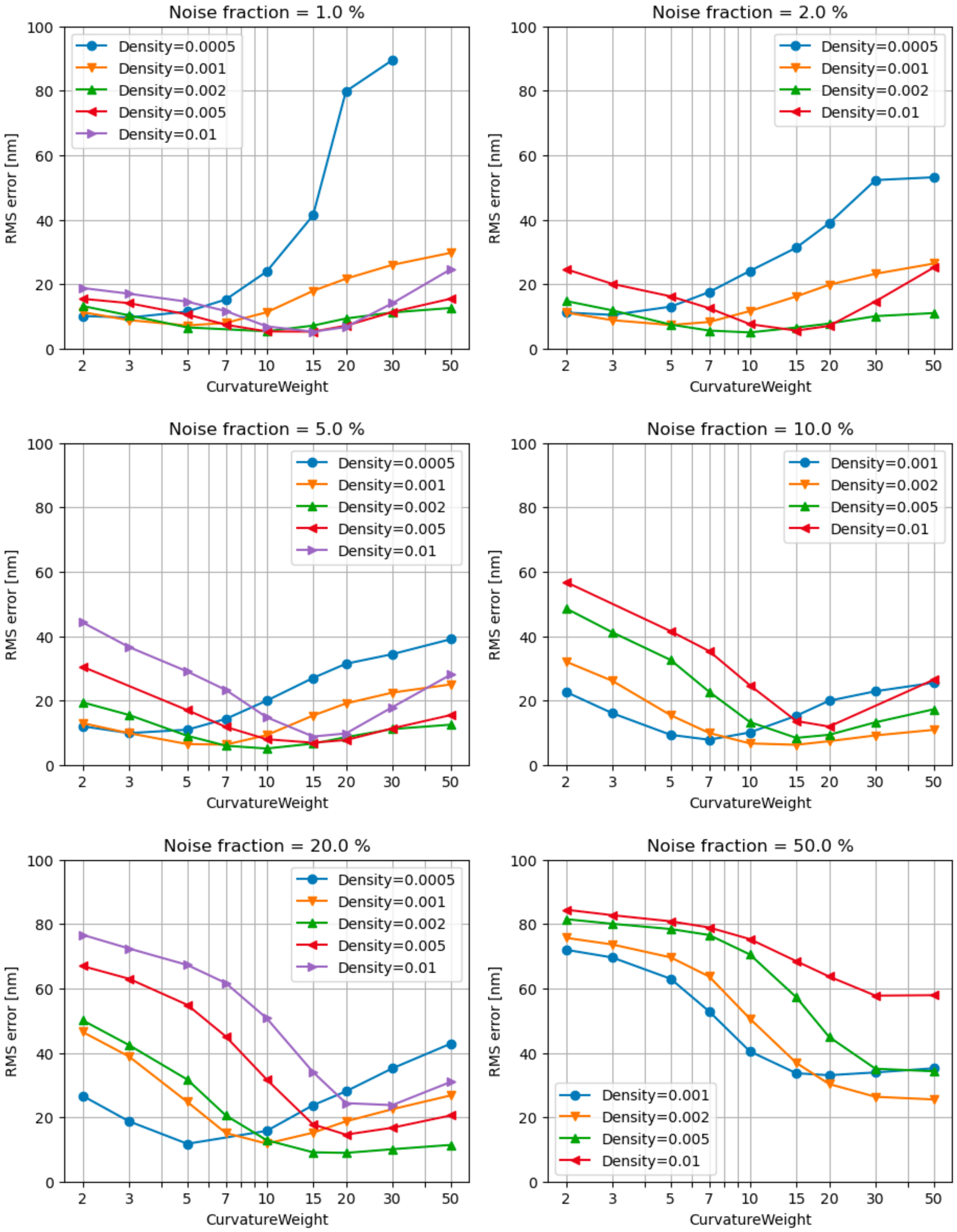

Figure S6: NanoWrap's sensitivity of fit performance to the chosen value of curvature weight at different values of structure density and background level. The optimal curvature weight increases with both increasing point density and background, but in each case there is a broad minimum.

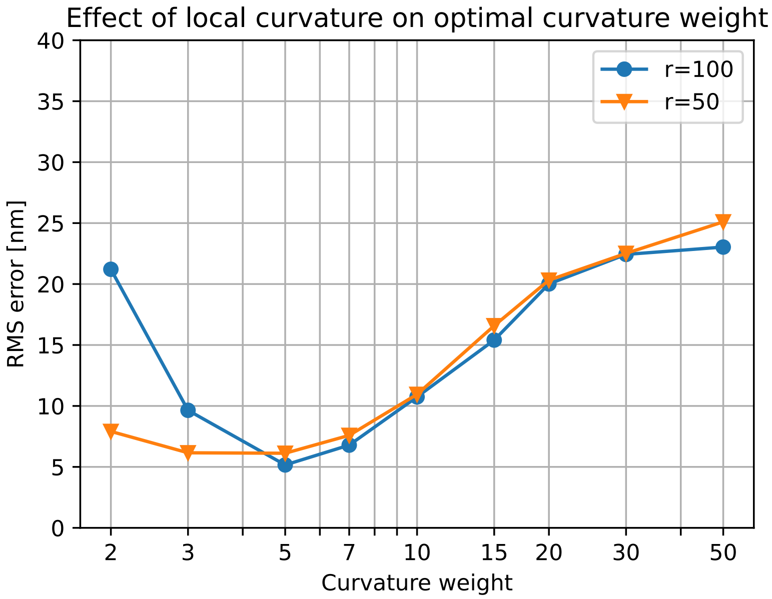

Figure S7: To test whether the optimal curvature weight depends on the underlying structure curvature we simulated a scaled version of the double-torus structure that had about twice the radius of curvature, whilst holding localisation precision, density, and noise levels constant in the middle of our range. There is only a very slight change in the optimal curvature weight, although the flatter structure shows a faster degradation in performance when the curvature weight is decreased below the optimum. This can be attributed to an additional regularising effect of the finite mesh size at small radii of curvature.

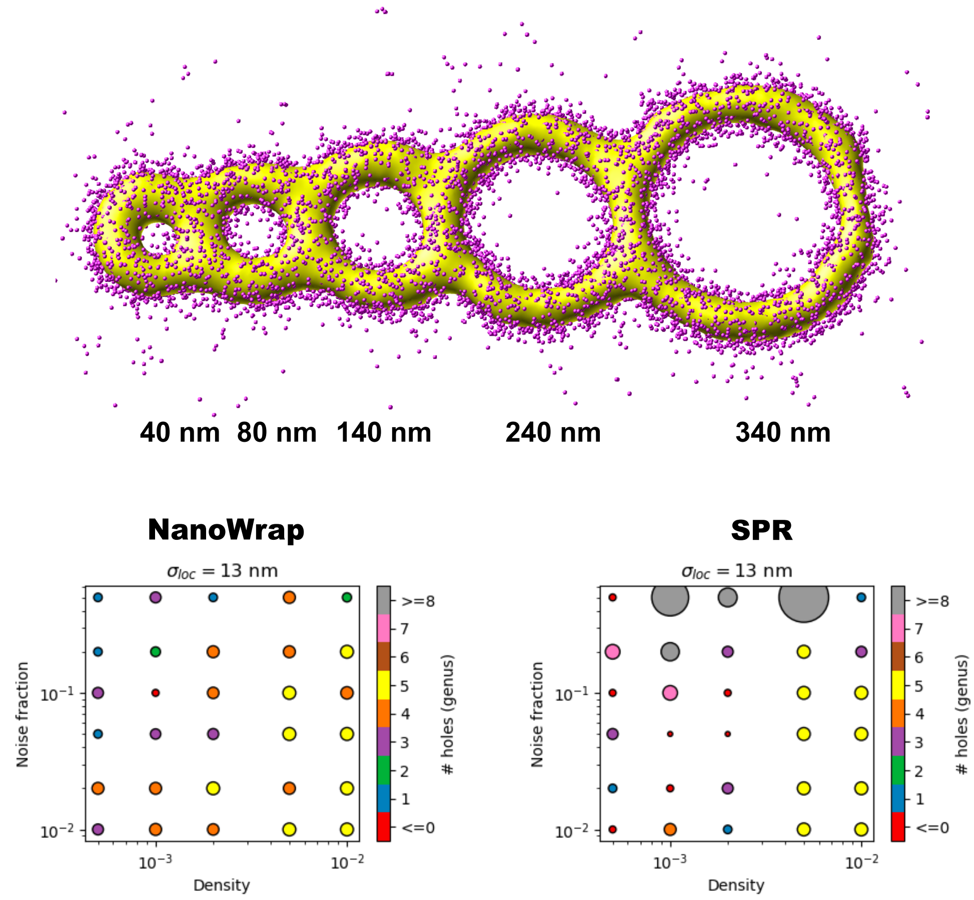

Figure S8: Ability of NanoWrap to correctly estimate the number of holes (genus) for a test structure using an automatically thresholded starting surface. Our ability to correctly estimate hole count is broadly comparable to SPR, but degrades a bit more gracefully at poor localization density. The genus estimation is very sensitive to the quality of the starting surface, and in the few cases where SPR correctly estimates the genus and we do not, the correct genus is easily obtained by manually tweaking the parameters for starting surface generation. This implies that there is still scope to improve the algorithm we use for intial surface estimation.

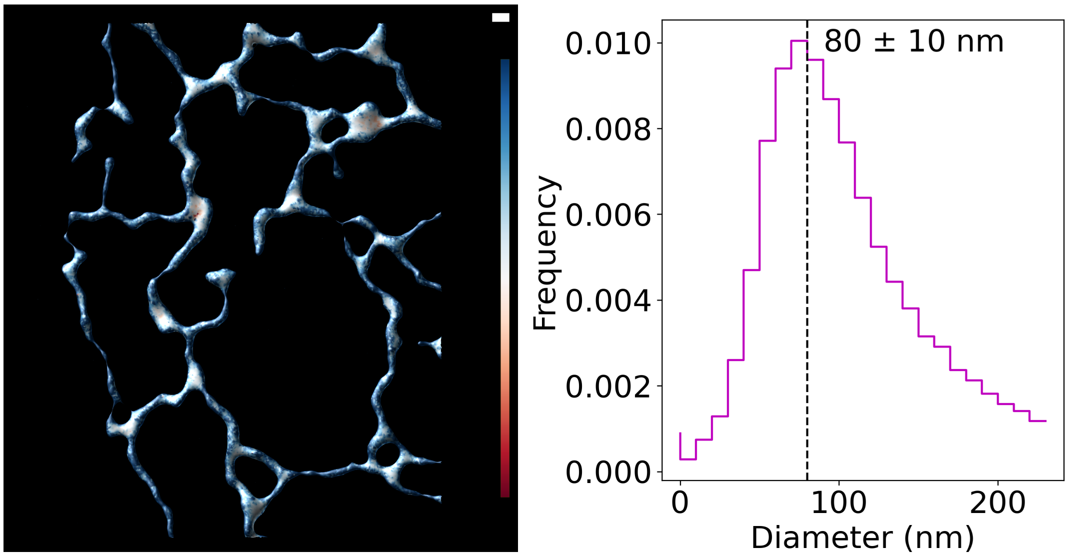

Figure S9: **Left,** Tubular ER from the lower left portion of Figure 3, colored by mean curvature. Lookup table: -0.01 to 0.01 nm^{-1}. Scale bar is 200 nm. **Right,** Histogram of $D = 2/k_{\max,l}$ where $k_{\max,l}$ is the maximal principal curvature of vertex l. The mean and standard error of the mean for the diameter distribution are reported.

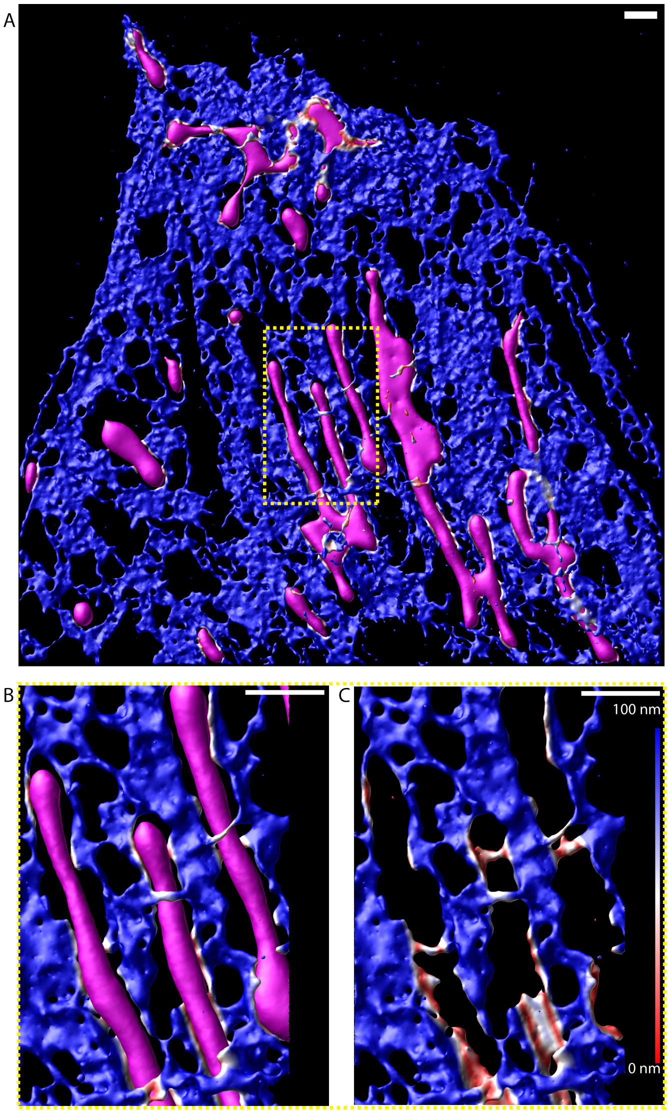

Figure S10: Distance between two surfaces. Surface generated from Sec61β (ER membrane) localizations (red-white-blue) and surface generated from TOMM20 (outer mitochondrial membrane) localizations (magenta). The Sec61β surface is colored by its distance from the TOMM20 surface (0 to 100 nm; red to blue). **A,** Full field of view, displaying both surfaces. **B,** Region of interest, showing both surfaces. **C,** The Sec61β surface alone. Scale bars are 1 μm.

USER GUIDE

This tutorial will walk you through using NanoWrap surface reconstruction within PYMEVisualize to create 3D surfaces based on a provided 3D point-cloud data set (`user-guide-data.hdf`). After completing this tutorial, you should be able to apply the same approach to fit your own 3D point-cloud data sets.

If you are unfamiliar with using PYMEVisualize, we strongly encourage you to first install PYME (see https://python-microscopy.org/doc/Installation/Installation.html) and go through the PYMEVisualize User Guide (https://python-microscopy.org/doc/PYMEVis.html) to understand the basics of the program before moving on to this tutorial (27).

If using the executable installers (recommended), the NanoWrap plugin should be automatically installed alongside PYMEVis. If this does not work, or for platforms where an executable installer is not available (currently linux and mac M1 native), PYME's installation instructions also cover how to set up Anaconda (https://anaconda.org/) and the Anaconda PYME environment. The NanoWrap plugin will then need to be installed manually following the instructions at https://github.com/python-microscopy/ch-shrinkwrap.

Verify Plugin Installation

Launch PYMEVis by either selecting from the start menu (windows) or by typing `PYMEVis` at the console (mac, linux) and hitting enter and confirm there is the menu option **Mesh → Shrinkwrap membrane surface** . Depending on how PYME was set up (executable vs manual conda-install) you might need to activate the conda environment - `conda activate PYME` - before the `PYMEVis` command is available. If the menu option is not visible, recheck the installation instructions at https://github.com/python-microscopy/ch-shrinkwrap.

Download and import tutorial data set

Download the data set `user-guide-data.hdf` found in the supplementary materials. Launch a PYMEVisualize window by entering `PYMEVis` into an Anaconda prompt with the PYME environment activated. Navigate to **File → Open** and select the `user-guide-data.hdf` file. To improve the visualization of the data, under the "Layers" side tab change "Method" to *pointsprites*, "Colour" to *z*, "Alpha" to *0.2*, and "Point size" to *10*. Your PYMEVisualize window should look identical to Figure S11 now.

Create initial isosurface

You can learn about PYMEVisualize isosurfaces and how to make them here. Once you're familiar with how to create an isosurface, use the parameters shown in Figure S12 to create an isosurface based on the `user-guide-data.hdf` points. Note that you can alternatively check "Auto threshold" to let NanoWrap estimate the ideal threshold density of the starting surface. See Supplementary Materials for a description of how automatic thresholding works. Once finished, you should see a 3D surface rendered with your points. Change "Method" to *wireframe* to more easily visualize how well the isosurface adheres to the point cloud. It should look something like Figure S13. At this point, it is a good idea to assess the overall accuracy of this initial isosurface. You can alter the parameters for the isosurface in the **Data Pipeline** tab on the left under **DualMarchingCubes**. It is typical to test several parameters before settling on the best options with "N points min" and "Threshold density" being the two major parameters requiring tweaking. It should be noted that this data set is a small cropped region of interest (ROI) from a much larger data set. It is highly recommended that you test parameters for the initial isosurface (and the fitted surface) on a small ROI of your data that has enough points to test the structure you're trying to create a surface from, but no more than that (about 20,000 points is ideal). The time required to create the surfaces scales roughly with the number of localizations, so testing parameters on large data sets is impractical. Once you establish which parameters are best with your smaller ROI, you can apply the same ones to the full data set. Learn how to create an ROI in PYMEVisualize here.

Fit surface with NanoWrap

Now that we have our initial isosurface, we can use it to create a fitted surface using NanoWrap. You will find this option under **Mesh → Shrinkwrap membrane surface**. The first window that pops up is requesting parameters to create an isosurface. If you haven't done so already make sure to create an isosurface because it is required for fitting. If you already have an isosurface, which you should if you are following this user guide, you can close this first window. The next window that pops up is requesting parameters you'd like to use for fitting. Refer to Table S2 for descriptions of what each parameter is and what typical values are for it. For this tutorial, use the parameters shown in S14 or check "Auto threshold". This process can take a minute or so. Check the Anaconda prompt for output to confirm it is running. Once finished, your surface should look similar to S15.

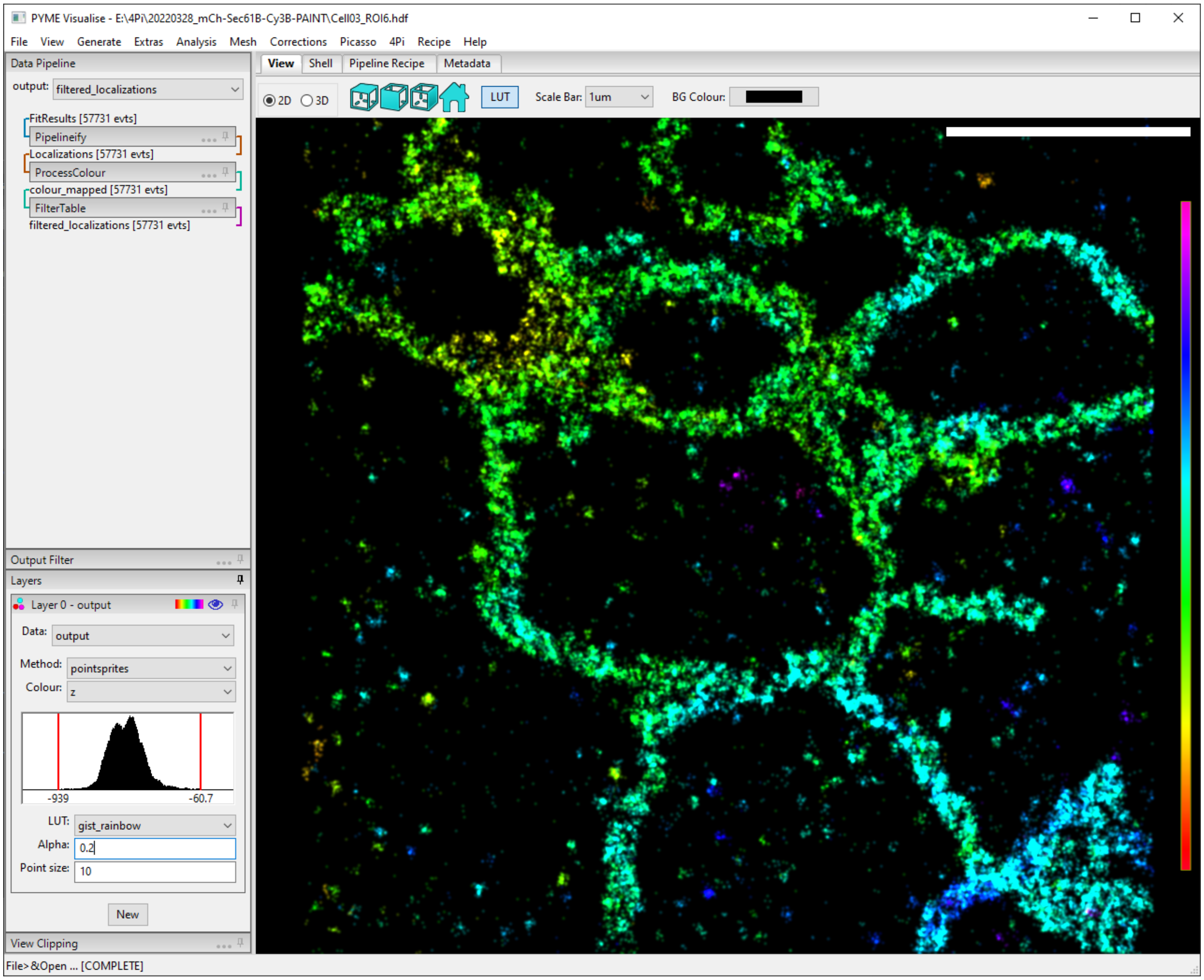

Figure S11: Data from `user-guide-data.hdf` displayed as 10 nm point sprites with 0.2 alpha. The points are colored by their location in the z-direction according to the lookup table on the right.

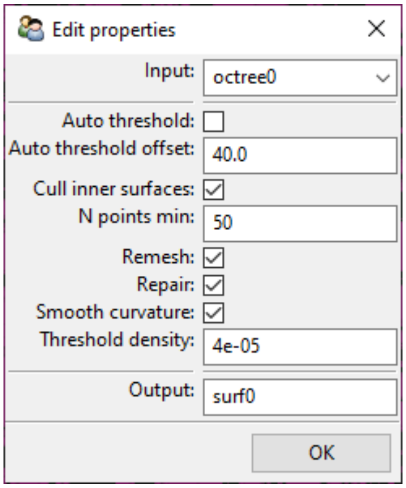

Figure S12: Ideal parameters to use to create an isosurface based on `user-guide-data.hdf`.

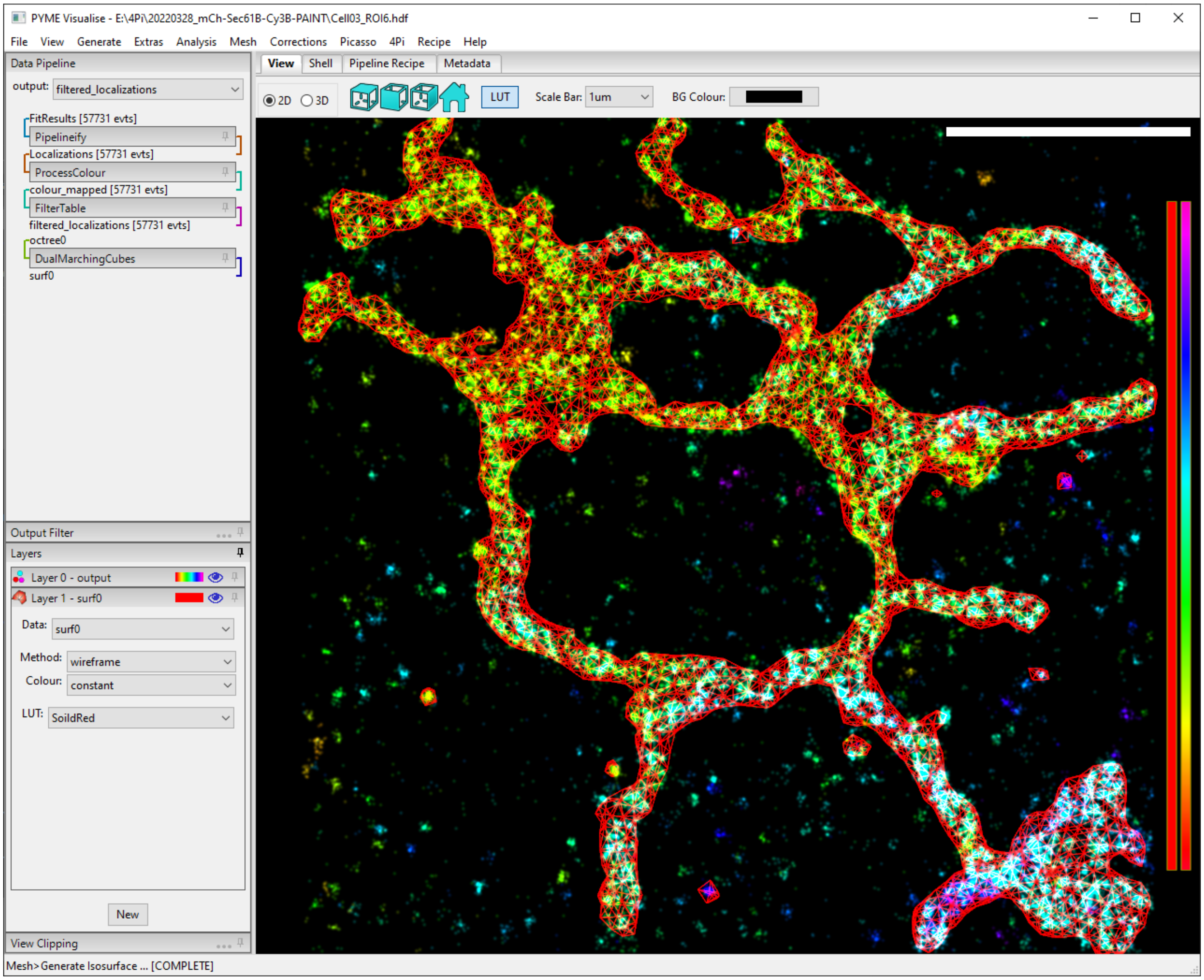

Figure S13: The isosurface generated using the parameters in S12. The isosurface is displayed using the *wireframe* method to make it easy to assess how well it fits the point cloud.

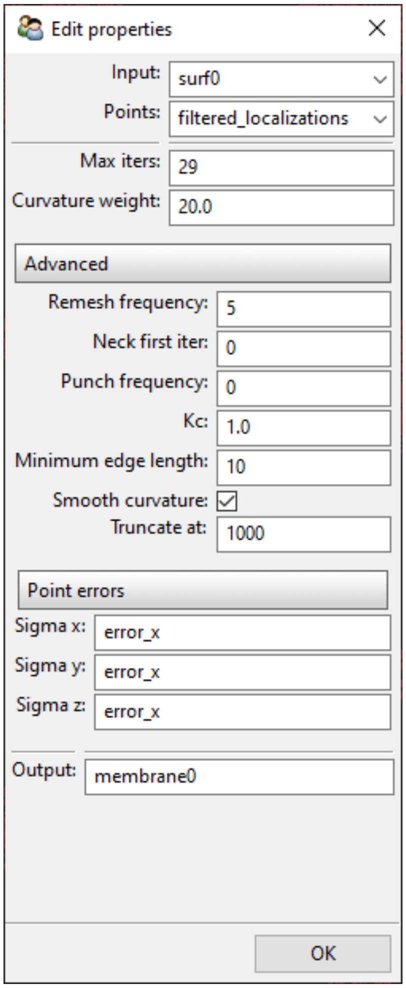

Figure S14: Ideal parameters to create a surface based on `user-guide-data.hdf`.

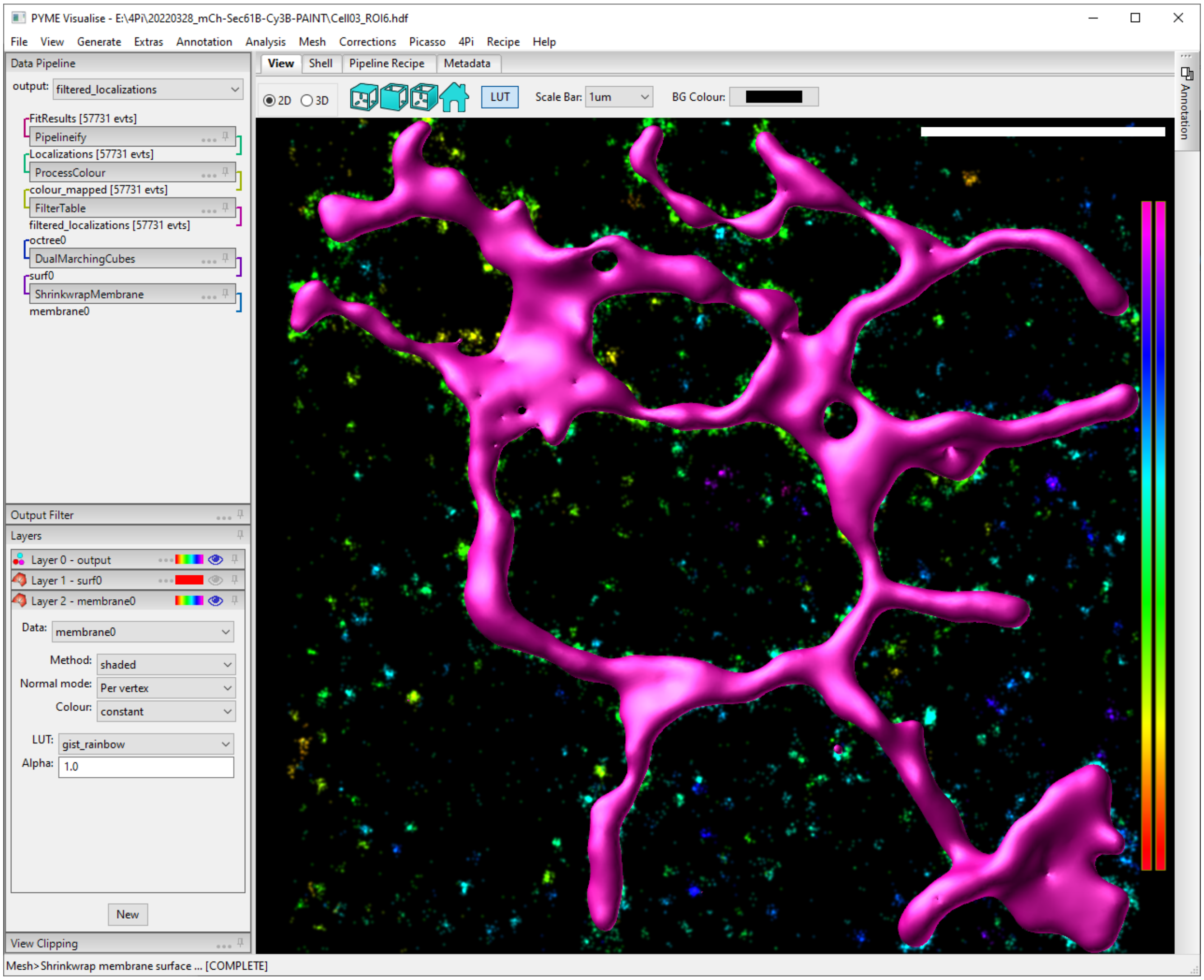

Figure S15: The surface generated using the parameters in S14.

Parameter	Description	Standard values
input	The data source containing the coarse isosurface.	`surf0`
Points	The data source containing the points to fit.	`filter_localizations`
Max iters	Maximum number of fitting iterations.	10 - 100
Curvature weight	The contribution of curvature (vs. point attraction force) to the fitting procedure. Higher values create smoother surfaces.	10 - 100
Remesh frequency	Remesh the isourface every N iterations. Helps keep the fitting numerically stable. Should be often.	5
Neck first iter	Every neck first iter iterations, check for and remove necks in the mesh.	9 (0 to not use necking)
neck_threshold_low	Vertices with Gaussian curvature below this threshold are necks.	-1.00E+03
neck_threshold_high	Vertices with Gaussian curvature above this threshold are necks.	1.00E+02
Punch frequency	Every punch frequency iterations, check for and add holes in regions of the mesh where there is a continuous empty area in between two "sides" of the mesh.	0
Kc	Lipid stiffness coefficient of membrane in eV (can be looked up in the literature).	0 - 1 (20k_bT)
Minimum edge length	Small length that the edges joining surface vertices can be. Smaller means the surface is more finely sampled. Setting it to 10 is usually sufficient. Setting it to -1 removes any limit.	10
Smooth curvature	Replace the fit curvature of a vertex with the average curvature of it and its neighbors	`True`
Truncate at	Stop after this many iterations no matter what. Useful for visualizing the behavior of a surface over time	1000
error_x	The variable in the points data source containing localization precision in the x-direction. If error is only known for one axis, supply it here and it will be assumed for all axes.	
error_y	The variable in the points data source containing localization precision in the y-direction.	
error_z	The variable in the points data source containing localization precision in the z-direction.	
output	The name of the data source that will contain the fit isosurface.	`membrane0`

Table S2: Descriptions and standard values for all parameters used in this paper's algorithm.

Additional tips for fitting surfaces

The following are tips for situations that did not come up in the tutorial, but are worth mentioning:

- An error that can occur when attempting to fit a surface is the "singular matrix" error. This simply means that the operation reached a point that was mathematically impossible to calculate. It is most often caused by several surfaces being present after creating an isosurface, especially small surfaces that are often capturing background localizations. Most of the time, this can be fixed be removing all but the largest surface present. To do so, enter the following line the the **Shell** tab in PYMEVisualize: `pipeline.dataSources['surf0'].keep_largest_connected_component()`.

NOTE `surf0` should be the name of **your** isosurface. By default it is `surf0`, but can be a different name for various reasons.

NOTE This solution clearly isn't always reasonable to use. For data from an ER protein, it is reasonable since the ER is continuous. However, data from a mitochondrial protein is not so reasonable since mitochondria are discontinuous. In the latter case, try to change the initial isosurface parameters, especially *N points min* and *Threshold density*, to resolve the error when fitting.

- If attempting to create several separate fit surfaces from multi-color data, make sure you are fitting to only the points belonging to the desired channel. By default, the algorithm uses `filtered_localizations` which includes all points in the data. To learn how to extract color channels from multi-color data in PYMEVisualize, please read the relevant documentation here.
